# Earthworms do not increase greenhouse gas emissions (CO_2_ and N_2_O) in an ecotron experiment simulating a three-crop rotation system

**DOI:** 10.1038/s41598-023-48765-3

**Published:** 2023-12-08

**Authors:** Oswaldo Forey, Joana Sauze, Clément Piel, Emmanuel S. Gritti, Sébastien Devidal, Abdelaziz Faez, Olivier Ravel, Johanne Nahmani, Laly Rouch, Manuel Blouin, Guénola Pérès, Yvan Capowiez, Jacques Roy, Alexandru Milcu

**Affiliations:** 1 Montpellier European Ecotron, Univ Montpellier, CNRS, Campus Baillarguet, 34980 Montferrier-Sur-Lez, France; 2 CEFE, Univ Montpellier, CNRS, EPHE, IRD, 34293 Montpellier, France; 3 Agroécologie, Institut Agro, INRAE, Univ. Bourgogne, Univ. Bourgogne Franche-Comté, 21000 Dijon, France; 4 UMR SAS INRAE Institut Agro Rennes-Angers, 65 Rue de Saint Brieuc, 35042 Rennes Cedex 10, France; 5 INRAE, UMR 1114 EMMAH, INRAE/Université d’Avignon, Site Agroparc, 84914 Avignon Cedex 09, France

**Keywords:** Agroecology, Climate-change ecology, Ecosystem ecology

## Abstract

Earthworms are known to stimulate soil greenhouse gas (GHG) emissions, but the majority of previous studies have used simplified model systems or lacked continuous high-frequency measurements. To address this, we conducted a 2-year study using large lysimeters (5 m^2^ area and 1.5 m soil depth) in an ecotron facility, continuously measuring ecosystem-level CO_2_, N_2_O, and H_2_O fluxes. We investigated the impact of endogeic and anecic earthworms on GHG emissions and ecosystem water use efficiency (WUE) in a simulated agricultural setting. Although we observed transient stimulations of carbon fluxes in the presence of earthworms, cumulative fluxes over the study indicated no significant increase in CO_2_ emissions. Endogeic earthworms reduced N_2_O emissions during the wheat culture (− 44.6%), but this effect was not sustained throughout the experiment. No consistent effects on ecosystem evapotranspiration or WUE were found. Our study suggests that earthworms do not significantly contribute to GHG emissions over a two-year period in experimental conditions that mimic an agricultural setting. These findings highlight the need for realistic experiments and continuous GHG measurements.

## Introduction

Earthworms are important decomposers in many ecosystems as they help to break down organic matter and release nutrients that can be used by plants and other organisms^1^. Thus, they are crucial for the functioning of many ecosystems and there is evidence that they play a vital role in supporting soil fertility and plant growth^2,3^, with the exception of ecosystems in which they are not native^4,5^. However, their activity can also lead sometimes to the release of greenhouse gases (GHG), such as carbon dioxide (CO_2_) and nitrous oxide (N_2_O)^6^. According to the latest meta-analysis^6^, earthworms can increase soil CO_2_ and N_2_O emissions by 33 and 42%, respectively. This is particularly concerning given the crucial role of soil in mitigating climate change through carbon (C) sequestration^7,8^ and N_2_O regulation^9,10^.

Earthworms are considered ecosystem engineers due to their ability to modify soil structure and interact with soil microorganisms and plants through their feeding, burrowing, and casting activities^11^. They can be divided into three ecological categories based on their feeding and burrowing habits: (1) anecic species that feed on fresh litter from the soil surface and create mainly permanent burrows, (2) epigeic species that live on the soil surface and feed on surface litter without creating permanent burrows, and (3) endogeic species that live and feed on mineral soil and associated organic matter below the surface, and that create non-permanent burrows without preferential orientation^12^. The impact of earthworms on greenhouse gas (GHG) emissions is known to vary with the earthworm ecotype, with anecic earthworms stimulating the GHG emissions the most. However, the full understanding of their effect on GHG remains elusive due to multiple contrasting reports published after the last meta-analysis, with multiple opposing reports challenging this general conclusion^13–23^.

A variety of factors, including the earthworm ecological category, the type of soil, the amount and type of organic matter present and the experimental setup have been reported to affect the earthworm effects on GHG emissions^6^. Earthworms can affect the soil CO_2_ emissions directly as the result of breaking down the soil and litter organic matter through digestive processes, releasing CO_2_ as a by-product, but also indirectly by incorporating plant residues into the soil, modulating the microbial-controlled decomposition of organic matter through changes in soil moisture dynamics, nutrient status, soil aggregation and CO_2_ diffusivity^1^. In addition to these effects that mainly stimulate the CO_2_ release from soils, earthworms have also been suggested to induce long-term stabilization of soil C in casts by enhancing the stabilization of C relative to mineralization^24^, but contrasting effects have also been found^25^. Concerning the earthworm impact on the N_2_O emissions, the proposed mechanisms are both direct, such as the stimulation of denitrifier activity in the earthworm gut due to favorable conditions for denitrifying bacteria such as anaerobic conditions, availability of nitrogen (N) and C at favorable moisture levels^26^, as well as indirect, including the stimulation of denitrifiers communities in the soil (as well as in the burrows, casts and middens) which can be further modulated by earthworms through incorporating plant residues in the soil and enhancing N and C mineralization^20^ as well as through burrowing effects on soil water infiltration and gas diffusivity^27^. These later two effects could also reduce N_2_O emissions^18^ if they lead to less anaerobic microsites and increased soil aeration, which is detrimental to denitrifiers^28^.

Considering the complexity and context-dependence of the mechanisms discussed above, it is not surprising that the literature contains a wide range of reports on the impacts of earthworms on GHG, which may be influenced by variations in experimental methodologies, and differences in environmental conditions. In this study, we addressed the need for realistic long-term experiments to evaluate the impact of earthworms on CO_2_ and N_2_O emissions; by realistic, we mean experiments that are simulating field-like conditions. Here this was achieved by using a relatively large replicated model system (5 m^2^ of surface, and 1.5 m depth lysimeters) simulating an agricultural context, and using an advanced controlled environment facility that has the capability to not only recreate environmental conditions but also to automatically and continuously measure the net ecosystem exchange of CO_2_, N_2_O and H_2_O fluxes^29,30^. We followed the impact of earthworm communities belonging to two ecological categories (endogeic and anecic separately) versus a control with reduced earthworm abundance and biomass over a two-year crop rotation with three crops (wheat–mustard–maize) and two fallow intercrops. Based on the lack of a significant stimulating effect of earthworms on GHG emissions in the field subset of last meta-analysis^6^, as well as in several other more recent studies^17–23^, we hypothesize that under simulated field-like conditions and in the presence of plants, higher anecic and endogeic earthworm biomass would not result in an increase in cumulative CO_2_ and N_2_O emissions or H_2_O loss as evapotranspiration, compared to a control with reduced earthworm biomass.

## Methods

### Macrocosms and soil

The experiment was conducted in the European Ecotron of Montpellier (Montferrier-sur-Lez, France, www.ecotron.cnrs.fr), an advanced controlled-environment experimental infrastructure developed by the Centre National de la Recherche Scientifique (CNRS) to study the response of ecosystems to global environmental changes. The Macrocosms platform used in this experiment consists of 12 identical and independent experimental units, each being composed of an ~ 30 m^3^ aboveground compartment enclosed by a highly transparent material to light and UV radiation (250 μm thick Teflon-FEP film, DuPont, USA) and a belowground compartment containing a 5 m^2^ stainless steel lysimeter hosting 14 t of soil (volume of ~ 7.5 m^3^); for additional information on the Macrocosms platform see Milcu et al.^31^ and Roy et al.^30^.

**Figure 1:**
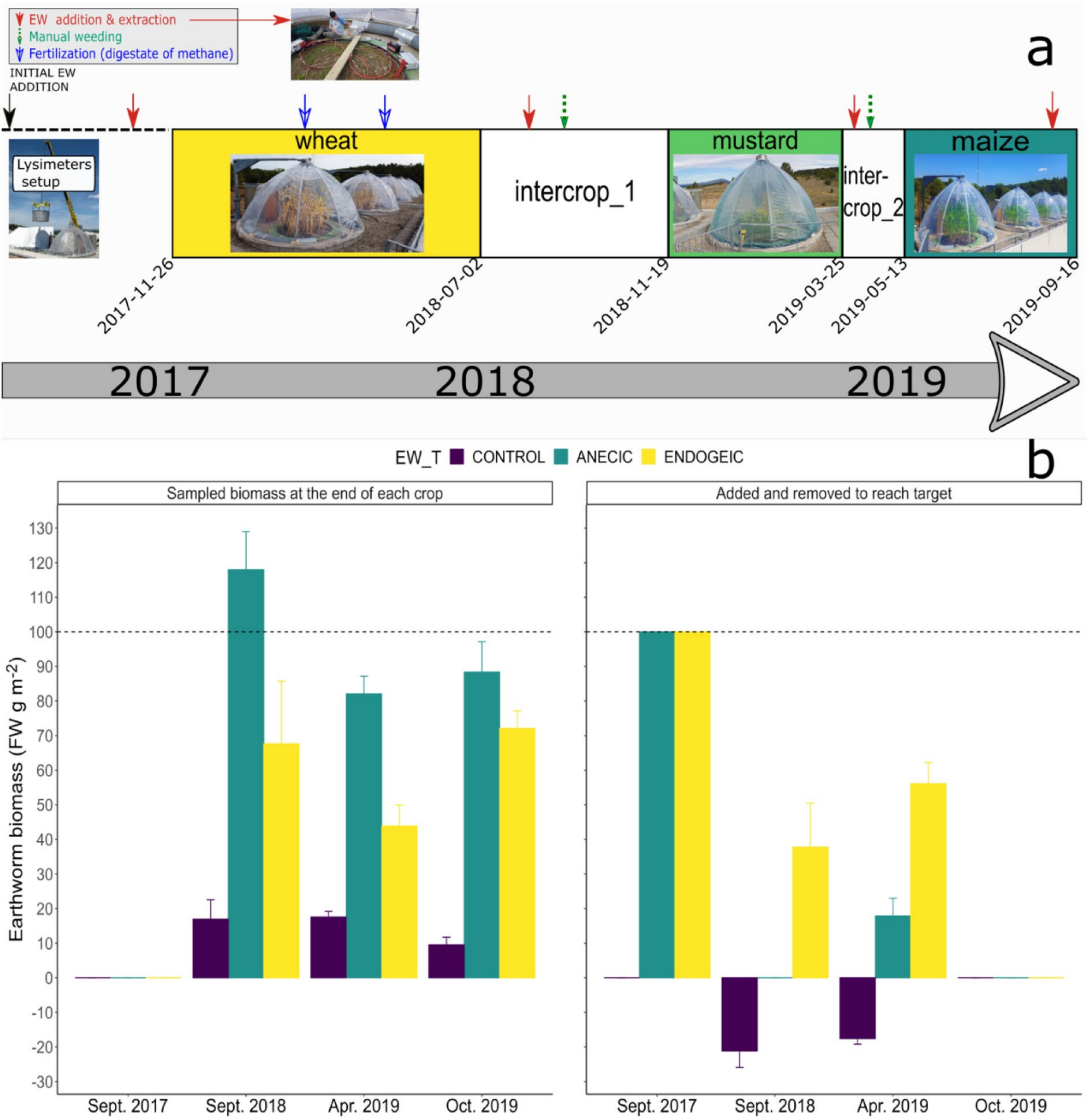
(**a**) Experimental timeline showing sowing and harvest/destruction dates along with earthworm (EW) extraction, manual weeding and fertilization events. The crop intervals are scaled to the number of days of each crop. (**b**) Earthworm fresh weight biomass was sampled at the end of each crop (left), and earthworms were added as needed (right) to achieve the minimum targeted earthworm biomass of 100 g FW m^2^ (horizontal dashed line) in the endogeic and anecic treatment combinations, while any sampled earthworms were removed from the control group. Data represent means ± SEM of four replicates.

The soil was excavated from field margins adjacent to the SOERE-PROs EFELE agricultural experimental site (Brittany, North West of France, 8° 05′ 35.9″ N, 1° 48′ 53.1″ W). According to the analyses performed by the Soil Analysis Laboratory, INRAE Arras, the upper 30 cm layer of this loamy soil (luvisol-redoxisol) is composed of 14.6% clay, 72.1% silt and 13.3% sand, with a pH of 6.14. It contains 1.5% total organic matter, 0.84% carbon, 0.1% nitrogen, with a C:N ratio of 8.4. The soil was excavated in three layers (0–0.3, 0.3–0.7, 0.7–1.5 m), transported to the ecotron where it was homogenised and reconstructed layer by layer in lysimeters outdoors. The aim of this process was to obtain the same soil density as in the original field, i.e., 1.35, 1.4 and 1.55 g cm^−3^ in the 0–0.3, 0.3–0.7 and 0.7–1.4 m soil layers respectively. The lysimeters were introduced in the Macrocosms platform in April 2017 (Fig. 1a) and left as a fallow until October 2017 when the first culture was sown after weeding any spontaneous vegetation and a superficial manual tillage (upper 5 cm) of the soil.

### Experimental setup—earthworm treatment and crop management

The experimental setup consisted in applying an earthworm treatment (with species from the Lumbricidae family), with three levels (1) anecic earthworms (A), (2) endogeic earthworms (E), and (3) a control (Ctr) with very low earthworm biomass. In the two levels with earthworms (A and E), three species for each ecological group were used: *Aporrectodea nocturna* Evans, *Lumbricus terrestris* L. and *Scherotheca gigas* Dugès. for the anecic level and *Aporrectodea chlorotica* Savigny, *Aporrectodea caliginosa* Savigny and *Aporrectodea icterica* Savigny for the endogeic level. Three species were used per earthworm ecotype in order to avoid identity effects; that is, to measure effects not attributable to a single earthworm species, thereby ensuring that the results can be generalized to the earthworm ecotype level, and not merely to the species level.

As the soil excavation, transport and recompacting to field density in lysimeters severely reduced the survival of living earthworms, at the onset of the experiment (April 2017), a total of 100 g FW m^−2^ earthworm biomass (with roughly equal biomass per species) originating from the EFELE site were inoculated/added in the lysimeters containing earthworms, and this biomass was kept as the minimal target earthworm biomass for the whole experiment (Fig. 1b). The target biomass is within the range of the earthworm biomass values at the EFELE site sampled in 2016, which ranged from 98 to 135 g FW m^−2^ (unpublished data).

To stimulate earthworm development and to prevent the excessive drying of the bare ground topsoil during the summer after the lysimeter filling, a total of 3.3 kg of dry plant residues (a mix of 0.25 kg of maize leaves, 1.36 kg of wheat straws and 1.72 kg of hay) was applied homogeneously at the surface of the soil at the end of June 2017. Earthworms were first sampled in October 2017 and thereafter at the end of each culture, and if the sampled earthworm biomass was lower than the target (100 g FW m^−2^), at each sampled point additional earthworms were added to reach the target biomass. Any earthworms sampled in the Ctr were removed, while the earthworms sampled in the A and E treatment levels were added back to their corresponding lysimeter following biomass evaluation. To ensure a maximal removal of earthworms, the Ctr lysimeters were subjected to dual sampling at each designated point, spaced several days apart. Earthworm sampling was conducted utilizing the non-invasive octet electric method^32^, which enabled sampling of a 1 m^2^ surface area for a duration of 50 min using a customized version of the octet device manufactured by Electrotechnik Schuller (Darmstadt, Germany). To achieve coverage of the entire 5 m^2^ surface area, five devices were simultaneously deployed (see Fig. 1a for the experimental timeline showing a picture of the devices and Fig. 1b showing the earthworm FW biomass sampled at each extraction).

The experiment simulated a three plant species crop rotation that is used at the reference EFELE site from where the soil originated, and which is composed of a succession of *Triticum aestivum*—*Sinapsis alba*—*Zea mays* (i.e., winter wheat–winter white mustard–maize). The seeds were provided by the INRAE EFELE site. No permissions or licenses were required and the collection of plant material complied with relevant legislation. The periods between the wheat and mustard as well as between mustard and maize are henceforth called intercrop_1 and intercrop_2, respectively (see Table S1 for the crop dates, sowing and harvesting information). During these periods, after the aboveground biomass was harvested, any unwanted plants/weeds were removed, and the soil surface was maintained as much as possible as bare ground, however some weed growth still occurred despite weeding (see Table S2 showing the weed biomass). Before sowing each crop, a manual and simplified superficial soil tillage (upper 5 cm only) was conducted to prepare a suitable seedbed for the next crop. All crops were manually sown, in rows for the wheat and maize crop, and broadcasted for the mustard crop; for the latter, the soil was pressed down with a rattle after sowing and 1.5 kg of wheat straw was added as top soil mulching. Fertilization was done only once during the wheat growing season through addition of methane digestate slurry supplied by Schiesslhof GbR farm (Neunburg, Bavaria). The digestate was applied using a watering can at the beginning of April (4.5 kg) and in mid-May 2018 (5 kg), dates corresponding to tillering and flowering/anthesis phenological stages, respectively, and amounting to an equivalent of 87 kg N ha^−1^ (Table S3 for the physico-chemical properties of the digestate). As in general the earthworm effects on GHG and plant growth vary with the fertilizer amendments^3,6^, we opted to only apply fertilizer only in one of the cultures in order to assess the impact of earthworms in conditions with and without fertilizer amendments. The experiment simulated the climatic conditions (air temperature, air humidity, and precipitation; see Figs. S1–S5) recorded in year 2017 at the EFELE experimental site and the conditions was recreated in the experimental years 2018–2020, with setpoints at 1 h intervals.

### ***Ecosystem CO***_***2***_***, N***_***2***_***O and water fluxes***

The CNRS Ecotron was designed to continuously measure CO_2_ net ecosystem exchange (NEE) by sequentially measuring the CO_2_ concentration at the inlet and outlet of each dome (every 12 min) using a multiplexer system coupled with two LI-7000 CO_2_/H_2_O analysers (LI-COR Biosciences, Lincoln, NE, USA). We used the Reichstein et al.^33^ C flux partitioning algorithm to estimate the daytime ecosystem respiration based on an exponential regression model^34^. This allowed for the estimation of ecosystem respiration over 24 h (Reco = Reco_night + Reco_day) and gross primary production (GPP = NEE_day − Reco_day).

Ecosystem-level N_2_O fluxes were measured continuously as an open system using a TILDAS Compact Single analyser (N_2_O Aerodyne Research, Inc., USA). The analyzer was coupled to a multiplexer system allowing N_2_O fluxes measurement every 72 min for each Macrocosm. Evapotranspiration (ET) was computed as the lysimeter weight difference between two consecutive days. Four shear beam load cells per lysimeter (CMI-C3, Precia-Molen, Privas CEDEx France), with an accuracy of ± 200 g, were used to measure the changes in mass. Ecosystem WUE was estimated as the ratio of GPP to ET derived from measurements by lysimeter weight changes over 24 h.

### Data treatment and statistical analyses

Data processing and statistical analyses were performed using R version 4.2.1 (R Development Core Team, 2015) in Rstudio version 2023.03.0 Build 386 (RStudio Team, 2015). Data was screened for outliers before statistical analyses and values that were lower or higher than 2× IQR for each replicate were considered to be outliers due to measurement errors or perturbations (e.g., when entering the domes, etc.). The C flux partitioning and gap-filling was performed within the “REddyProc (v. 1.3.2)” package^35^. Missing values from the N_2_O time series were replaced with the predicted values from a loess regression, with a 0.05 span.

We conducted three distinct but complementary statistical analyses, each aimed at discerning specific aspects of the ecosystem fluxes: (1) an analysis of the treatment effect on weekly averaged fluxes to capture potential differences in temporal dynamics, (2) a per crop analysis of cumulated ecosystem fluxes and (3) a whole crop sequence cumulated analysis including the data from the whole experiment.

As maintaining completely earthworm-free controls and identical levels of earthworm biomass in large lysimeters during an extensive experiment is practically impossible, we controlled for the low earthworm presence in the controls (Fig. 1b) by consistently introducing sampled earthworm biomass (EW_BM) at the end of each culture as a covariable in the statistical analyses along the earthworm treatment (EW_T; see “Supplementary Information” section for more details on statistical analyses).

## Results

### Wheat crop

**Figure 2:**
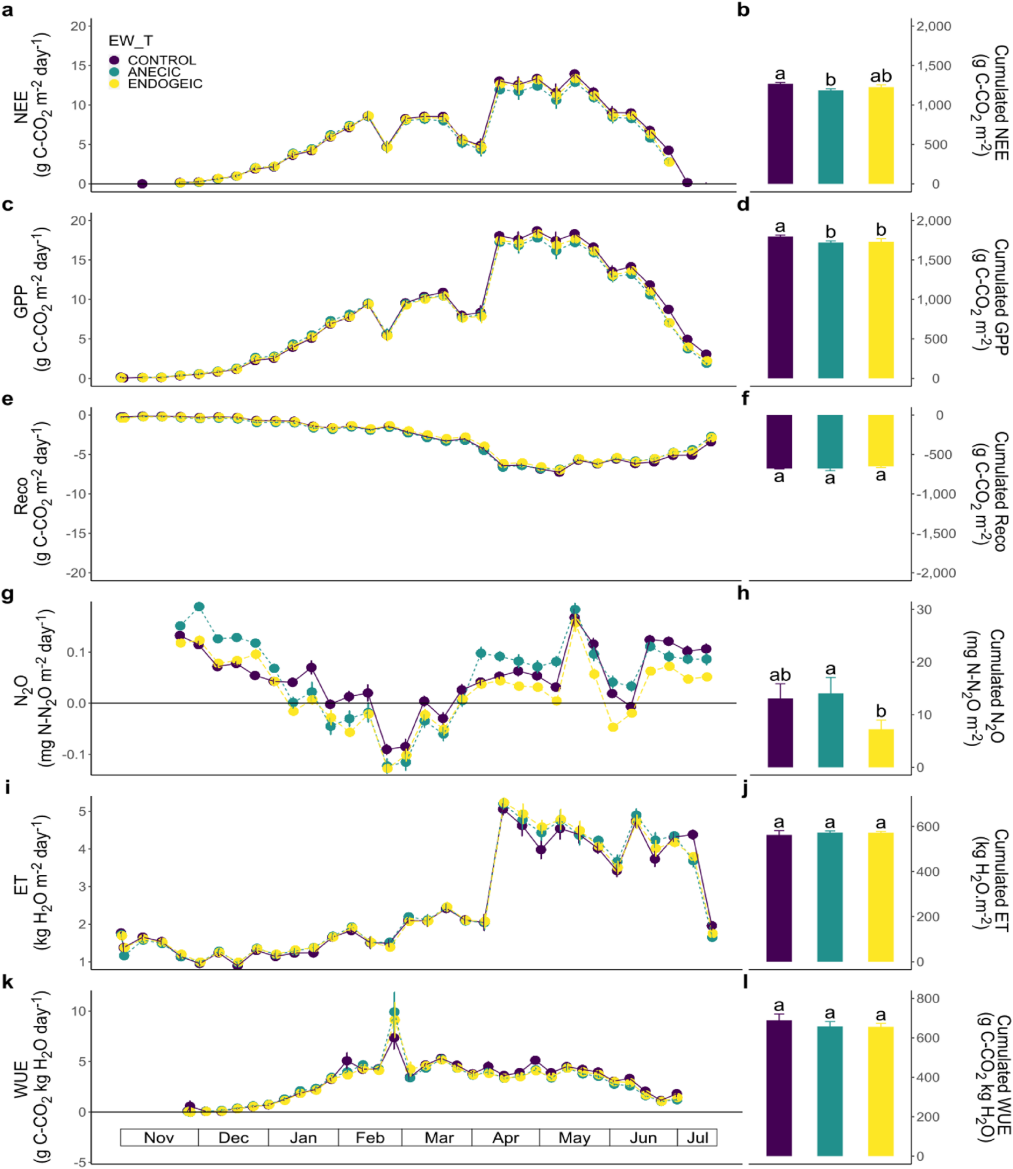
Weekly dynamics (left) and cumulative fluxes (right) of carbon, N_2_O and water fluxes as affected by the earthworm treatments in the wheat crop. (**a**, **b**) NEE. (**c**, **d**) GPP. (**e**, **f**) Reco. (**g**, **h**) N_2_O. (**i**, **j**) ET. (**k**, **l**) WUE. Data represent means ± SEM of four replicates. Different letters above bars denote significant differences between means according to Tukey’s HSD post-hoc test.

NEE followed the wheat growing stages (Fig. 2a) as shown by the Week explanatory variable retained in the minimum adequate model (P-value < 0.001; Table 1), but no significant effect of the earthworm treatment (EW_T) nor of the amount of earthworm biomass (EW_BM) was found (Table 1). Cumulative NEE fluxes over the whole wheat crop (Fig. 2b; Table 1) showed a marginally significant effect of EW_T, with lower cumulative values (− 6.44%, Table 1; Fig. 2b) in the anecic earthworm treatment level relative to control (P-value = 0.086). Unlike NEE, weekly GPP fluxes (Fig. 2c) showed an EW_BM effect, GPP fluxes slightly increasing with EW_BM (P-value = 0.027; Table 1), and a strong Week effect (P-value < 0.001; Table 2). The cumulative GPP flux was found to be slightly increasing with EW_BM (irrespective of the earthworm ecological category; Table 1), it was also marginally significantly lower in the anecic (− 4.26%) and endogeic (− 3.66%) earthworm treatment levels relative to control (P-value = 0.065; Table 1; Fig. 2d). The later result combined with the simultaneous positive EW_BM effect on GPP indicates that higher biomass within each treatment level also led to a slight increase in GPP. A similar relationship between EW_BM and EW_T was occasionally observed in other response variables.

**Table 1:** Minimal adequate models (F-values) for (1) weekly time series as affected by the sampling week (Week), earthworm biomass (EW_B), treatment (EW_T) and their interactions, and (2) cumulative emissions as affected by the earthworm biomass (EW_B) and treatment (EW_T) in the wheat crop.

Weekly time series						
Source	NEE	GPP	Reco	N_2_O	ET	WUE
Week	**F**_**32/352**_ = 895.45***	**F**_**32/352**_ = 1330.22***	**F**_**32/288**_ = 642.24***	**F**_**28/308**_ = 30.79***	**F**_**32/352**_ = 299.31***	**F**_**32/352**_ = 1624.52***
EW_BM	NA	**F**_**1/10**_ = 6.68***	NA	NA	NA	NA
EW_T	NA	NA	**F**_**2/9**_ = 5.19***	F2/9 = 2.54	NA	NA
EW_T:Week	NA	NA	**F**_**64/288**_ = 1.73***	NA	NA	NA
Cumulative						
EW_BM	NA	**F**_**1/8**_ = 7.99***	**F**_**1/8**_ = 41.41***	F_1/8_ = 1.59	NA	NA
EW_T	F_2/9_ = 3.38^+^	F_2/8_ = 3.91^+^	**F**_**2/8**_ = 12.18***	F_2/8_ = 3.06	NA	NA

“NA” stands for non-applicable.

***P < 0.001; **P < 0.01; *P < 0.05; ^+^P < 0.1.

Significant values are in bold.

**Table 2:** Minimal adequate models (F-values) for (1) weekly time series as affected by the sampling week (Week), earthworm biomass (EW_B), treatment (EW_T) and their interactions, and (2) cumulative emissions as affected by the earthworm biomass (EW_B) and treatment (EW_T) in the mustard crop.

Weekly time series						
Source	*NEE*	GPP	Reco	N_2_O	ET	WUE
Week	**F**_**18/198**_ = 322.35***	**F**_**18/162**_ = 1326.67***	**F**_**18/162**_ = 280.5***	**F**_**18/162**_ = 85.55***	**F**_**18/198**_ = 307.84***	**F**_**18/198**_ = 214.94***
EW_BM	F_1/8_ = 0.65	F_1/8_ = 0.1	NA	NA	NA	F_1/10_ = 2.37
EW_T	F_2/8_ = 1.84	F_2/8_ = 0.96	F_2/9_ = 2.12	F_2/9_ = 2.71	NA	NA
EW_T:Week	NA	**F**_**36/162**_ = 1.76***	F_36/162_ = 0.71	**F**_**36/162**_ = 2***	NA	NA
Cumulative						
EW_BM	**F**_**1/8**_ = 40.52***	**F**_**1/8**_ = 18.47***	NA	NA	F_1/10_ = 3.78^+^	NA
EW_T	F_2/8_ = 1.38	F_2/8_ = 0.62	NA	NA	NA	**F**_**2/9**_ = 4.66***

“NA” stands for non-applicable.

***P < 0.001; **P < 0.01; *P < 0.05; ^+^P < 0.1.

Significant values are in bold.

Reco weekly emissions increased with wheat development until the beginning of May and slowly decreased thereafter with the senescence of the plants until the harvest (Fig. 2e). Reco weekly fluxes were significantly affected by the EW_T × Week interaction (P-value = 0.001; Table 1), with several weeks where the anecic earthworms stimulated Reco at the beginning of the crop, however this changed in the middle of the crop where Reco values were higher in the endogeic treatment level relative to control and during the last 4 weeks of the experiment where Reco values were higher both in the anecic and endogeic earthworm treatment levels relative to control. The cumulative Reco fluxes generally increased with earthworm biomass (P-value < 0.001; Table 1) and was also affected by the EW_T (P-value = 0.004; Table 2), with significantly lower fitted model parameter estimates for the endogeic treatment level relative to control (− 4.4%); however, the Tukey’s HSD test used in Fig. 2f does not capture this difference.

The analyses of the weekly dynamics of N_2_O emissions showed a significant Week effect (P-value < 0.001; Fig. 2g; Table 1) and a stimulation of emissions after the addition of fertilizer (digestate of methanisation in April and May). A tendency for an EW_T effect (P-value = 0.133; Table 1; Fig. 2g) was also found, with the endogeic earthworms marginally reducing N_2_O emissions (− 19.8%) relative to control. Analysis of the cumulative N_2_O confirmed that the N_2_O emissions were statistically marginally significantly lower in the endogeic earthworm treatment level relative to control (P-value = 0.109), however the effect size was notable (− 44.6%; Table 1, Fig. 2h).

ET and WUE showed no statistically significant effects of EW_T or EW_BM (Table 1, Fig. 2i–l).

### Mustard crop

**Figure 3:**
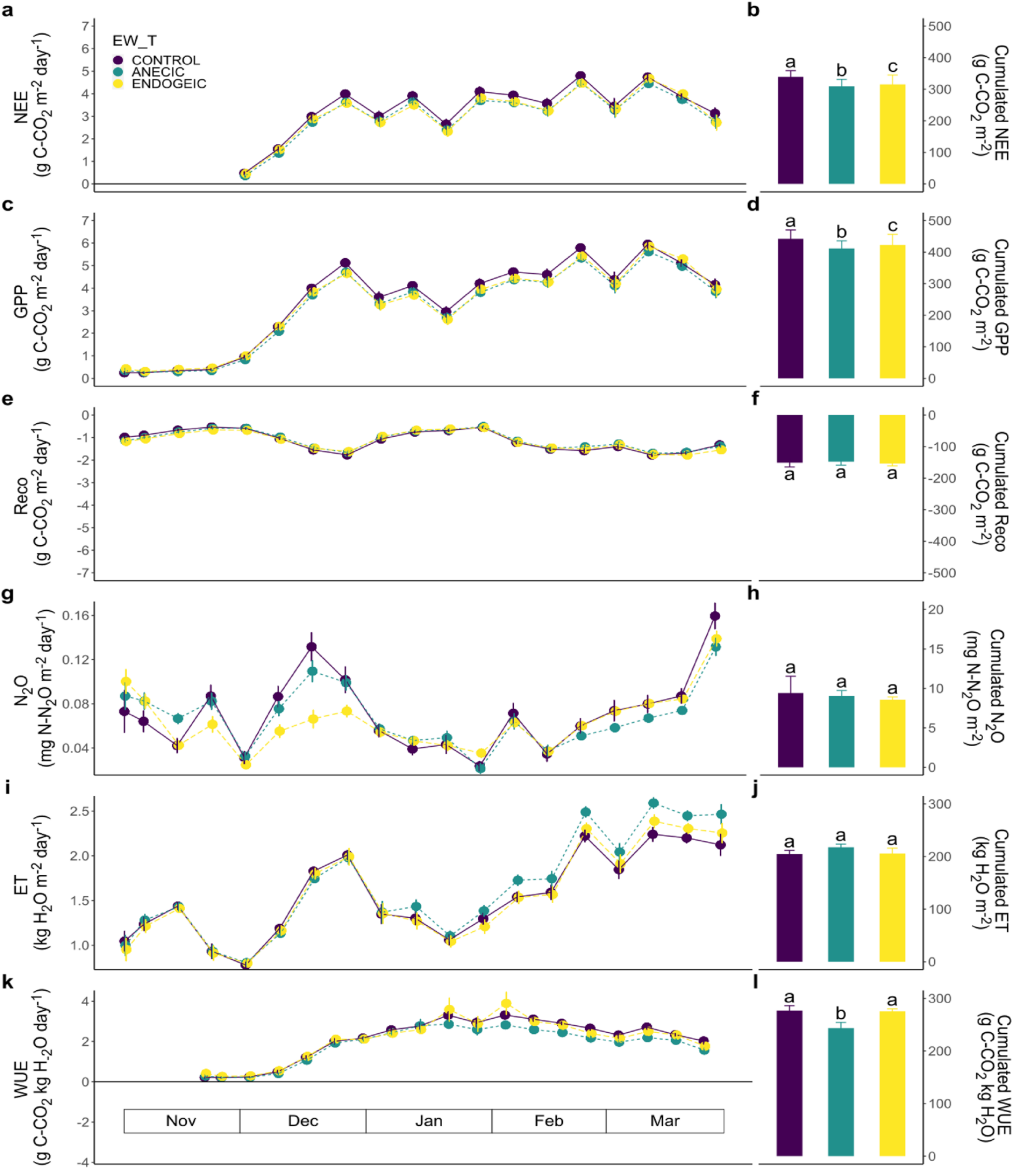
Weekly dynamics (Left, lines) and cumulative fluxes (Right, bars) of carbon, N_2_O and water fluxes as affected by the earthworm treatments in the mustard crop. (**a**, **b**) NEE. (**c**, **d**) GPP. (**e**, **f**) Reco. (**g**, **h**) N_2_O. (**i**, **j**) ET. (**k**, **l**) WUE. Data represent means ± SEM of four replicates. Different letters above bars denote significant differences between means according to Tukey’s HSD post-hoc test.

The weekly NEE (Fig. 3a) only varied with time (P-value < 0.001) and no significant effect of EW_BM nor EW_T was found (Table 2). However, the cumulative NEE emissions were found to increase with EW_BM (P-value < 0.001; Table 2, Fig. 3b).

Weekly GPP fluxes (Fig. 3c) were significantly affected by the EW_T × Week interaction (P-value = 0.009; Table 2), with several weeks where the GPP was significantly lower in the endogeic treatment level in the middle of the growing season. The cumulative GPP fluxes were also found to increase with EW_BM (P-value = 0.003; Table 2, Fig. 3d).

Reco weekly emissions fluctuated with the changes in environmental conditions and the development of the mustard canopy and showed transient tendencies of higher Reco under anecic and endogeic treatment levels in the first 4 weeks of the culture (Fig. 3e). The cumulative Reco fluxes showed no statistically significant effects either of EW_T or EW_BM (Table 2; Fig. 3f).

The weekly N_2_O emissions (Fig. 3g) were significantly affected by the EW_T × Week interaction (P-value < 0.001; Table 2), with marginally higher N_2_O the last 2 weeks of December in the endogeic treatment level relative to control. However, these effects proved to be transient, as the cumulative N_2_O fluxes showed no statistically significant effects of EW_BM nor EW_T (Table 2; Fig. 3h).

Weekly ET fluxes followed the crop development and increased progressively from about 1 kg m^−2^ day^−1^ from the start of the mustard growing season to ~ 2 kg m^−2^ day^−1^ before the crop harvest (Fig. 3i), and only a significant Week effect was found (P-value < 0.001; Table 2). Cumulative ET fluxes were not affected by EW_BM nor EW_T (Table 2; Fig. 3j).

WUE weekly means varied with Week (P-value < 0.001; Table 2, Fig. 3k) and were affected by the EW_T with statistically significantly lower (− 12%) values in the anecic treatment level relative to control (P-value = 0.041; Table 2; Fig. 3l).

### Maize crop

**Figure 4:**
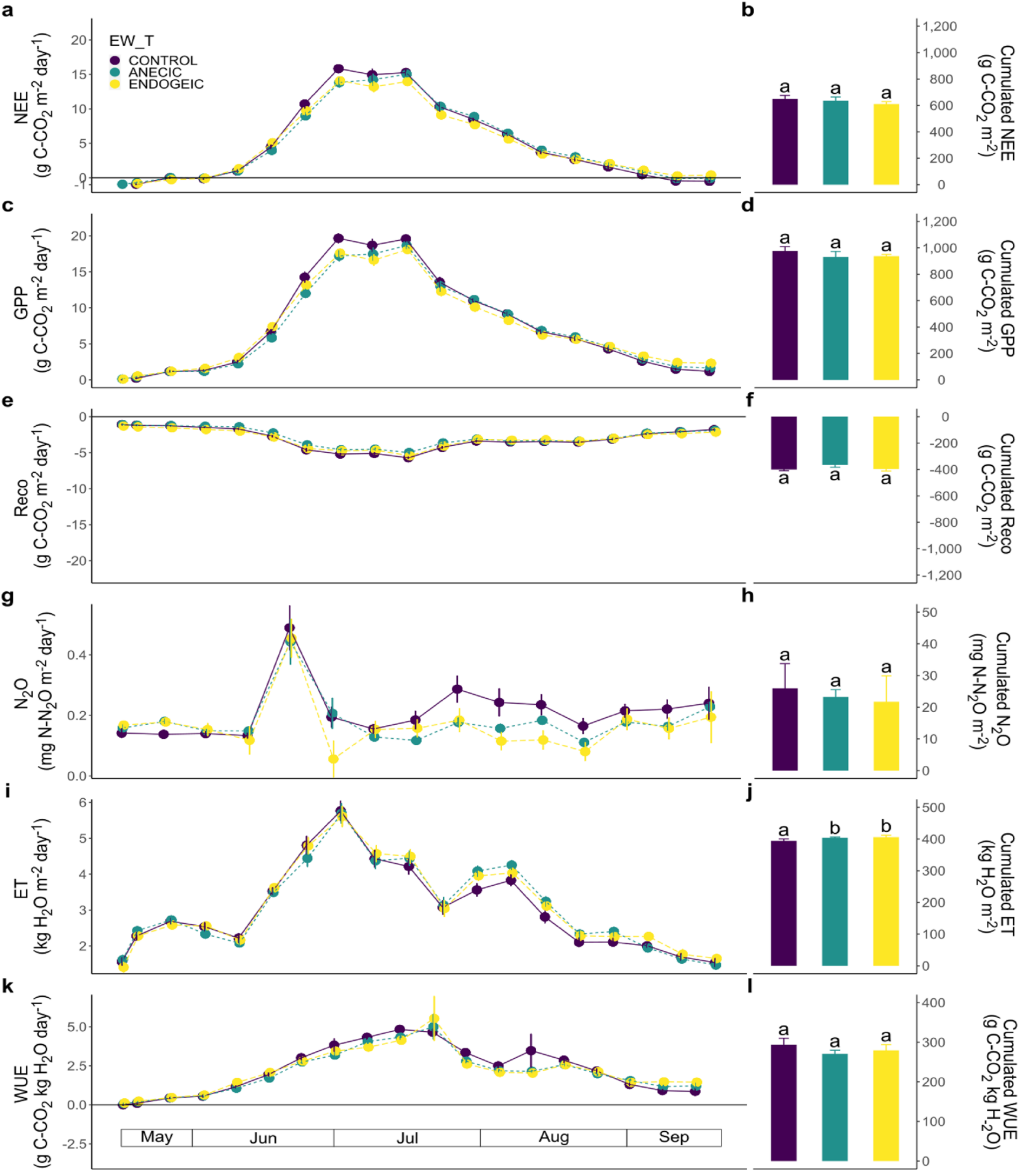
Weekly dynamics (Left, lines) and cumulative fluxes (Right, bars) of carbon, N_2_O and water fluxes as affected by the earthworm treatments in the maize crop. (**a**, **b**) NEE. (**c**, **d**) GPP. (**e**, **f**) Reco. (**g**, **h**) N_2_O. (**i**, **j**) ET. (**k**, **l**) WUE. Data represent means ± SEM of four replicates. Different letters above bars denote significant differences between means according to Tukey’s HSD post-hoc test.

The NEE weekly fluxes were influenced by the EW_T × Week interaction (P-value = 0.002; Table 3, Fig. 4a), with higher NEE the second week of June 2019 and in the last 2 weeks of the experiment for the endogeic treatment level relative to control. The cumulative NEE emissions were not influenced by EW_BM or EW_T (Table 3; Fig. 4b). Weekly GPP fluxes showed similar pattern and effects as NEE (Fig. 4c), and were influenced by the EW_T × Week interaction (P-value = 0.001; Table 3), with transient stimulation (e.g., weeks 21, 24 and 38) or dampening (week 32; second week of August) of GPP fluxes by the endogeic earthworms relative to the control (Fig. 4c). The cumulative GPP were not influenced by the earthworm treatment (Table 3; Fig. 4d).

**Table 3:** Minimal adequate models (F-values) for (1) weekly time series as affected by the sampling week (Week), earthworm biomass (EW_B), treatment (EW_T) and their interactions, and (2) cumulative emissions as affected by the earthworm biomass (EW_B) and treatment (EW_T) in the maize crop.

Weekly time series						
Source	NEE	GPP	Reco	N_2_O	ET	WUE
Week	**F**_**18/162**_ = 235.87***	**F**_**18/162**_ = 231.97***	**F**_**18/198**_ = 94.35***	**F**_**14/154**_ = 11.84***	**F**_**18/198**_ = 623.3***	**F**_**18/198**_ = 1719.96***
EW_BM	NA	NA	NA	NA	**F**_**1/8**_ = 3439.95***	**F**_**1/8**_ = 1457.34***
EW_T	F_2/9_ = 1.26	F_2/9_ = 2.75	NA	NA	**F**_**2/8**_ = 395.68***	**F**_**2/8**_ = 47.09***
EW_T:Week	**F**_**36/162**_ = 1.96***	**F**_**36/162**_ = 2.12***	NA	NA	NA	NA
Cumulative						
EW_BM	NA	F_1/10_ = 2.75	NA	NA	**F**_**1/8**_ = 34.02***	NA
EW_T	NA	NA	NA	NA	F_2/8_ = 1.51	NA

“NA” stands for non-applicable.

***P < 0.001; **P < 0.01; *P < 0.05.

Significant values are in bold.

Reco weekly emissions followed quite similar dynamics to NEE and GPP, following maize development, however with a less steep decrease in absolute value during maize senescence (Fig. 4e). Reco weekly fluxes were not significantly affected by EW_BM or EW_T, but they significantly varied with Week (P-value < 0.001; Table 3). Cumulative Reco fluxes showed no statistically significant effects of EW_BM or EW_T (Table 3; Fig. 4f).

The weekly N_2_O emissions were only significantly influenced by Week (P-value < 0.001; Table 3, Fig. 4g) and the cumulative N_2_O fluxes showed no EW_BM or EW_T statistically significant effect (Table 3; Fig. 4h).

Weekly ET fluxes followed the crop development (Fig. 4i) and were significantly affected by Week (P-value < 0.001), EW_T (P-value < 0.001) and EW_B (P-value < 0.001; Table 3). The EW_T effect indicated higher ET under both the anecic and endogeic treatment combination whereas the EW_BM indicated a decrease of ET with EW_BM. Cumulative ET fluxes were not affected by the EW_T, but overall ET decreased with EW_BM (P-value < 0.001; Table 3, Fig. 4j).

Weekly WUE rates were significantly affected by Week (P-value < 0.001; Table 3, Fig. 4k), EW_BM and EW_T. It increased with EW_BM (P-value < 0.001; Table 3) and was lower in the endogeic and anecic earthworms relative to control (P-value < 0.001; Table 3. The cumulative WUE showed no statistically significant effect of the earthworm treatment (Table 3; Fig. 4l).

Refer to the Supplementary Information file (Figs. S6 and S7) for the weekly dynamics and cumulative fluxes obtained during the two intercropping periods (i.e., between wheat and mustard, and between mustard and maize) as well as the associated statistical analyses (Tables S4 and S5).

### Whole crop rotation

**Figure 5:**
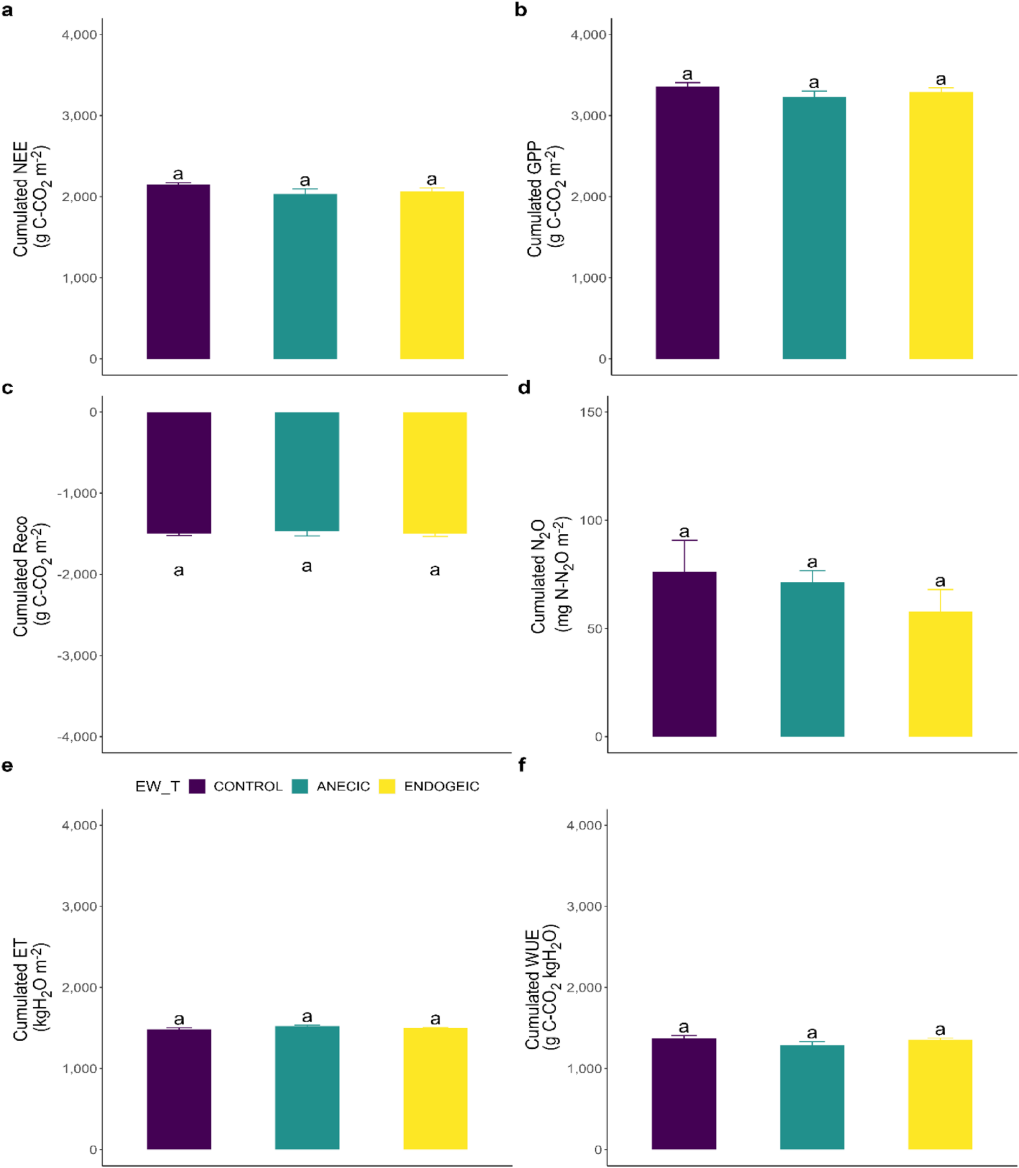
Cumulative fluxes of carbon, N_2_O and water fluxes as affected by the earthworm treatments over the whole crop rotation. (**a**) NEE. (**b**) GPP. (**c**) Reco. (**d**) N_2_O. (**e**) ET. (**f**) WUE. Data represent fluxes cumulated over 640 days ± SEM of four replicates. Different letters above bars denote significant differences between means according to Tukey’s HSD post-hoc test.

The cumulative values over the whole crop rotation including the three main cultures and the two intercrop periods (see “Supplementary Information” section on the results of the two intercrop periods) and approximately 24 months of experimentation are shown for each variable on Fig. 5. Of all six response variables, none showed any significant EW_T nor EW_BM effects.

## Discussion

### Earthworm effects on carbon fluxes (NEE, GPP, Reco)

In line with our hypothesis, our findings suggest that the earthworm treatment had only a minor and transient effect on carbon fluxes during certain periods of the experiment. However, when considering the entire two-year period, neither the earthworm treatment nor the earthworm biomass had a significant impact on carbon fluxes. Although Lubbers et al.^6^ reported a 33% increase in soil CO_2_ emissions in the presence of earthworms, it remains unclear whether this translates into ecosystem-level carbon losses. Our results suggest that this is not the case. However, it should be noted that our measurements were taken at the ecosystem-level and included both plant and soil fluxes, which differ from those reported by Lubbers et al.^6^ that only considered soil CO_2_ emissions. Nonetheless, we observed periods of short-term stimulation of ecosystem respiration (Reco) during experimental periods with minimal plant contribution to CO_2_ fluxes (e.g., before and immediately after sowing or during intercrop periods after aboveground biomass harvest). For instance, we observed such transient stimulations during the first few weeks of mustard growth and during intercrop_2 (between mustard and maize). However, these temporary increases in soil CO_2_ emissions did not result in ecosystem-level carbon losses over the course of our experiment. Although there are numerous studies that do not align with our findings, our results are consistent with several literature reports based on field experiments that demonstrate the short-lived impacts of earthworms on soil CO_2_ emissions. For example, Borken et al.^36^ conducted an earthworm inoculation experiment in a beech forest and observed that the presence of earthworms (*L. terrestris*) led to a 16–28% increase in CO_2_ emissions during the initial 3–4 weeks, but subsequently, the emissions were reduced after 11 weeks. Similarly, Schindler Wessels et al.^37^ in a 2-year field experiment (corn agroecosystem), found that CO_2_ emissions were stimulated by earthworms, but only in the second year during the period going from June to August and not during the first year due to a severe drought. In a more recent field study (upland rice), John et al.^38^ showed that earthworms did not affect CO_2_ emissions over the whole rice growing season but that they did have an effect depending on the rice growth stage. Last, but not least, the results are in line with the findings of Ganault et al.^19^ performed on the same soil and using a subset of the same earthworm species in a mesocosm experiment with plants, which showed no significant effect of earthworms on soil CO_2_ emissions.

### ***Earthworm effects on N***_***2***_***O emissions***

Consistent with our hypothesis, we did not observe a stimulation of N_2_O emissions by earthworms when considering the cumulative emissions across the entire experiment. Moreover, the presence endogeic earthworms let to a statistically marginal reduction of N_2_O emissions in the wheat (− 48.6%) and intercrop_1 period (i.e., between wheat and mustard) and a similar trend was noted for the mustard and maize crops. Our findings are in agreement with several other studies showing a reduction of N_2_O emissions by earthworms^17–23^. In particular, using soil from the same site the one used in our experiment, Ganault et al.^19^ reported a significant reduction in N_2_O emissions in mesocosm experiments when the endogeic species *A. icterica* was present. It's worth noting that in our experiment the inhibitory impact of endogeic earthworms on N_2_O emissions was more prominent (both statistically and in terms of effect size) following fertilizer application during the wheat cultivation and subsequent intercrop, but less apparent during other crop cycles without nitrogen fertilization. These results suggest that this phenomenon may be more pronounced under conditions conducive to N_2_O emissions, such as higher soil N availability as was also found by Kan et al.^39^. While due to the size of the experimental system no data on soil porosity was included in this study, it is possible that a mechanism similar to the one proposed by Ganault et al.^19^ could be at play, whereby endogeic earthworms promote soil aeration, leading to reduced denitrification and N_2_O emissions, as also suggested by several studies^20,22^. The aforementioned effect is expected to be comparatively weaker for anecic earthworm species, since although they are known to create larger burrows, the total number of burrows and macropores generated per unit volume of soil is anticipated to be lower^40^.

### ***Earthworm effects on H***_***2***_***O fluxes (ET, WUE)***

Similar to the C fluxes, while punctual effects of our earthworm treatment on ET were observed in some periods, no significant impact on cumulated fluxes was found over the entire experimental period. However, it is worth noting that during maize, intercrop_1 and intercrop_2, ET significantly decreased with earthworm biomass. Since ET is the combination of soil evaporation and plant transpiration, the decrease in ET with EW_BM in intercrop periods with minimal vegetation suggests that the effect mainly resulted from reduced evaporation from the topsoil. This reduction is likely due to the faster infiltration of water into the deeper soil layers, consistent with previous studies^41^. The presence of anecic earthworms in the mustard treatment also resulted in a 12% decrease in WUE, while the presence of endogeic earthworms led to a 28% increase during intercrop_2. While a mechanistic explanation of these effects is not straightforward, we can speculate that the reduction of WUE in the anecic treatment could be attributed to their feeding behavior. Anecic earthworms are known to be able to consume small seeds, such as mustard seeds, which could have impacted the density of the established plants^42^, thus reducing the GPP.

### The ecotron results in the context of field and very simplified microcosm experiments

Our findings contradict Lubbers et al.’s overall conclusion^6^, but align with their field experiment subset, which reported a non-significant effect. Several subsequent studies also reported an enhancement of N_2_O emissions by earthworms. Without being exhaustive, these studies are lacking continuous measurements of fluxes^43^, and are either conducted in extremely small and artificial setups without plants^15,16,44,45^ or in semi-field conditions by installing pots^14^ or small separating enclosures/containers in the soil in the field^39^, often with much smaller surface areas than in our experiment and with modified precipitation regimes. Of the two studies approaching the most to field-like conditions, the results are inconclusive. Lubbers et al.^14^ reported that earthworm reduced the N_2_O emissions in spring but with an opposite effected in autumn (and with the caveat that the flux measurements were always performed 24 h after watering). On the other hand, Kan et al.^39^ reported variable effects depending on whether straw was added to the enclosures (with lower emissions in the earthworm treatment if straw was added).

A possible explanation for the contrasting results relative to the latest meta-analysis is that the results obtained from highly simplified model systems lacking important features of field conditions such as plants, natural light, deep soil, surface litter layer, and a watering protocol that allows for the earthworm burrows affect water drainage and soil moisture fluctuations, cannot be generalized to field conditions. Other common drawbacks of many experiments include limited duration and the use of only point measurements of fluxes, which may fail to capture the full range of phenomena and processes in the system. These conjectures are supported by experimental results that included two main naturally occurring factors were found to mitigate earthworm mediated CO_2_ and N_2_O emissions, namely: (1) growing plants and (2) soil water content fluctuations due to drainage or drying and rewetting cycles^17–21^. Plants, as primary producers, play a crucial role in biogeochemical cycles because they determine the amount and quality of carbon that enters the soil system^46^. Plants also compete with microbes for nitrogen acquisition^47^, decrease soil water content through transpiration, and modify soil porosity through root growth, which can alter the dominant processes that produce N_2_O emissions (nitrification, denitrification, and nitrate ammonification)^28,48^. This is in line with the results from a mesocosm experiment with plants and earthworms^19^, which found that the presence of plants lowered N_2_O emissions by 19.8%, in correlation with a 43% and 20% decrease in nitrate and ammonium respectively.

Studies have shown that soil moisture can explain up to 95% of GHG emissions^49^. The availability of oxygen, nitrates, ammonium, and carbon in the soil is determined by the moisture content, which in turn affects the activity of microorganisms. Furthermore, anoxic conditions under high soil water content can stimulate N_2_O emissions, primarily through the process of denitrification, while nitrification is more likely to occur in aerobic conditions with unsaturated soil moisture. Naturally occurring fluctuations in soil moisture and drying-rewetting cycles, can affect the proportion of denitrified nitrogen that is converted to N_2_O or N_2_, ultimately modulating the N_2_O/N_2_ ratio that is emitted into the atmosphere^50,51^. Therefore, experimental setups aiming for constant soil moisture, as used in many laboratory incubations, or that are measuring fluxes only after watering, are likely to lead to biased conclusions.

### Caveats and limits of this experiment

While our study benefits from the advantages of a realistic long-term and large-scale ecosystem sample in controlled environment conditions, there are several caveats that must be acknowledged. Because of the inherent trade-off in ecotron facilities between their advanced capabilities and limited number of experimental units, we were unable to test multiple soils or include a combination of endogeic and anecic earthworms. As a consequence, our results should be extrapolated with caution beyond the scope of our experimental setup. The relatively low level of replication (n = 4) may also limit the statistical power necessary to detect effects with a lower effect size, although we argue that the frequent and continuous measurements available in the ecotron facility partially compensate for this limitation. Another limitation that we share with some of the previous experiments that aimed to manipulate earthworm biomass in field-like conditions^14,52^ is that, despite our efforts to achieve an earthworm-free control by removing all sampled earthworms during two extractions before each culture, some earthworms were still present in the control; estimated earthworm biomass in the controls at the end of the culture ranged from 9.56 g FW m^−2^ in maize to 21.16 g FW m^−2^ in wheat. However, to account for this effect, the sampled earthworm biomass at the end of each culture was used a covariable in all statistical analyses.

## Conclusion and perspectives

Based on a 2-year experiment in an advanced controlled environment facility^30^, specifically designed for continuous measurements of ecosystem fluxes over replicated large model ecosystems simulating agricultural management, our findings indicate that earthworms do not stimulate the ecosystem-level emissions of greenhouse gases (CO_2_ and N_2_O), and that in certain conditions, endogeic earthworms may even reduce N_2_O emissions. However, our results are in line with those of Lubbers et al.^6^ in that we found transient stimulations of soil CO_2_ and N_2_O emissions under certain conditions (in the first weeks after sowing and during the intercrop periods), although these effects were offset by periods of low emissions over the duration of the entire experiment. In conclusion, our study highlights the importance of realistic experimental setups that allow for continuous high-frequency measurements and emphasized the importance of experimental designs that include plants and allow for the earthworm engineering effect on soil water status and aeration to take place in a realistic way. Drawing on our findings and an expanding body of research demonstrating that under realistic conditions, earthworms do not result in elevated greenhouse gas emissions^18,19,38^, we recommend updating the meta-analysis performed by Lubbers et al.^6^. This includes incorporating more recent studies and assigning additional weight to studies that adhere to more realistic experimental conditions.

### Supplementary Information

Supplementary Information.

## Data Availability

The datasets generated during this study as well as an R Markdown file documenting the statistical analyses are available at 10.5061/dryad.mgqnk9955.

## References

[1] Edwards CA, Arancon NQ (2022). Biology and Ecology of Earthworms.

[2] Scheu S (2003). Effects of earthworms on plant growth: Patterns and perspectives: The 7th international symposium on earthworm ecology · Cardiff · Wales · 2002. Pedobiologia.

[3] van Groenigen JW (2014). Earthworms increase plant production: A meta-analysis. Sci. Rep..

[4] Ferlian O (2018). Invasive earthworms erode soil biodiversity: a meta-analysis. J. Anim. Ecol..

[5] Resner K (2015). Invasive earthworms deplete key soil inorganic nutrients (Ca, Mg, K, and P) in a northern hardwood forest. Ecosystems.

[6] Lubbers IM (2013). Greenhouse-gas emissions from soils increased by earthworms. Nat. Clim. Change.

[7] Lal R (2004). Soil carbon sequestration impacts on global climate change and food security—PubMed. Sci. New Ser..

[8] Soussana J-F (2019). Matching policy and science: Rationale for the ‘4 per 1000—Soils for food security and climate’ initiative. Soil Tillage Res..

[9] Butterbach-Bahl K, Baggs EM, Dannenmann M, Kiese R, Zechmeister-Boltenstern S (2013). Nitrous oxide emissions from soils: How well do we understand the processes and their controls?. Philos. Trans. R. Soc. B Biol. Sci..

[10] Hu H-W, Chen D, He J-Z (2015). Microbial regulation of terrestrial nitrous oxide formation: Understanding the biological pathways for prediction of emission rates. FEMS Microbiol. Rev..

[11] Jouquet P, Dauber J, Lagerlöf J, Lavelle P, Lepage M (2006). Soil invertebrates as ecosystem engineers: Intended and accidental effects on soil and feedback loops. Appl. Soil Ecol..

[12] Bouché, M. B. Strategies lombriciennes. *Ecol. Bull.* 122–132 (1977).

[13] Gong X (2021). Cattle manure biochar and earthworm interactively affected CO_2_ and N_2_O emissions in agricultural and forest soils: Observation of a distinct difference. Front. Environ. Sci. Eng..

[14] Lubbers IM, González EL, Hummelink EWJ, Van Groenigen JW (2013). Earthworms can increase nitrous oxide emissions from managed grassland: A field study. Agric. Ecosyst. Environ..

[15] Maslov M, Astaykina A, Pozdnyakov L (2022). Earthworm *Lumbricus terrestris* contributes nitrous oxide emission from temperate agricultural soil regardless of applied mineral nitrogen fertilizer doses. Agronomy.

[16] Schorpp Q (2016). Influence of *Lumbricus terrestris* and *Folsomia candida* on N_2_O formation pathways in two different soils—With particular focus on N_2_ emissions. Rapid Commun. Mass Spectrom..

[17] Chen C, Whalen JK, Guo X (2014). Earthworms reduce soil nitrous oxide emissions during drying and rewetting cycles. Soil Biol. Biochem..

[18] Ejack L (2021). Earthworms did not increase long-term nitrous oxide fluxes in perennial forage and riparian buffer ecosystems. Pedobiologia.

[19] Ganault, P. *et al.* No evidence that earthworms increase soil greenhouse gas emissions (CO_2_ and N_2_O) in the presence of plants and soil moisture fluctuation. 10.21203/rs.3.rs-2162558/v1 (2022).

[20] Kuiper I, de Deyn GB, Thakur MP, van Groenigen JW (2013). Soil invertebrate fauna affect N_2_O emissions from soil. Glob. Change Biol..

[21] Law MMS, Lai DYF (2021). Impacts of wetting-drying cycles on short-term carbon and nitrogen dynamics in Amynthas earthworm casts. Pedosphere.

[22] Lubbers IM, Berg MP, De Deyn GB, van der Putten WH, van Groenigen JW (2019). Soil fauna diversity increases CO_2_ but suppresses N_2_O emissions from soil. Glob. Change Biol..

[23] Zhang W (2013). Earthworms facilitate carbon sequestration through unequal amplification of carbon stabilization compared with mineralization. Nat. Commun..

[24] Zhang W (2013). Root distribution and interactions in jujube tree/wheat agroforestry system. Agrofor. Syst..

[25] Lubbers IM, Pulleman MM, Van Groenigen JW (2017). Can earthworms simultaneously enhance decomposition and stabilization of plant residue carbon?. Soil Biol. Biochem..

[26] Matthies C, Griesshammer A, Schmittroth M, Drake HL (1999). Evidence for involvement of gut-associated denitrifying bacteria in emission of nitrous oxide (N_2_O) by earthworms obtained from garden and forest soils. Appl. Environ. Microbiol..

[27] Rizhiya E (2007). Earthworm activity as a determinant for N_2_O emission from crop residue. Soil Biol. Biochem..

[28] Baggs EM (2011). Soil microbial sources of nitrous oxide: Recent advances in knowledge, emerging challenges and future direction. Curr. Opin. Environ. Sustain..

[29] Roy J (2016). Elevated CO_2_ maintains grassland net carbon uptake under a future heat and drought extreme. Proc. Natl. Acad. Sci..

[30] Roy J (2021). Ecotrons: Powerful and versatile ecosystem analysers for ecology, agronomy and environmental science. Glob. Change Biol..

[31] Milcu A (2014). Functional diversity of leaf nitrogen concentrations drives grassland carbon fluxes. Ecol. Lett..

[32] Schmidt O (2001). Appraisal of the electrical octet method for estimating earthworm populations in arable land. Ann. Appl. Biol..

[33] Reichstein M (2005). On the separation of net ecosystem exchange into assimilation and ecosystem respiration: Review and improved algorithm. Glob. Change Biol..

[34] Lloyd J, Taylor JA (1994). On the temperature dependence of soil respiration. Funct. Ecol..

[35] Wutzler T (2018). Basic and extensible post-processing of eddy covariance flux data with REddyProc. Biogeosciences.

[36] Borken W, Gründel S, Beese F (2000). Potential contribution of *Lumbricus terrestris* L. to carbon dioxide, methane and nitrous oxide fluxes from a forest soil. Biol. Fertil. Soils.

[37] Schindler Wessells ML, Bohlen PJ, Mccartney DA, Subler S, Edwards CA (1997). Earthworm effects on soil respiration in corn agroecosystems receiving different nutrient inputs. Soil Biol. Biochem..

[38] John K (2020). Earthworms offset straw-induced increase of greenhouse gas emission in upland rice production. Sci. Total Environ..

[39] Kan Z-R (2023). Straw incorporation interacting with earthworms mitigates N_2_O emissions from upland soil in a rice-wheat rotation system. Sci. Total Environ..

[40] Capowiez Y, Gilbert F, Vallat A, Poggiale J-C, Bonzom J-M (2021). Depth distribution of soil organic matter and burrowing activity of earthworms—Mesocosm study using X-ray tomography and luminophores. Biol. Fertil. Soils.

[41] Capowiez Y (2021). Using the ecosystem engineer concept to test the functional effects of a decrease in earthworm abundance due to an historic metal pollution gradient. Appl. Soil Ecol..

[42] Milcu A, Schumacher J, Scheu S (2006). Earthworms (*Lumbricus terrestris*) affect plant seedling recruitment and microhabitat heterogeneity. Funct. Ecol..

[43] Nieminen M, Hurme T, Mikola J, Regina K, Nuutinen V (2015). Impact of earthworm *Lumbricus terrestris* living sites on the greenhouse gas balance of no-till arable soil. Biogeosciences.

[44] Marhan S, Auber J, Poll C (2015). Additive effects of earthworms, nitrogen-rich litter and elevated soil temperature on N_2_O emission and nitrate leaching from an arable soil. Appl. Soil Ecol..

[45] Zhu X (2018). Interactions between earthworms and mesofauna affect CO_2_ and N_2_O emissions from soils under long-term conservation tillage. Geoderma.

[46] Chapin FS, Matson PA, Vitousek P (2011). Principles of Terrestrial Ecosystem Ecology.

[47] Hodge A, Robinson D, Fitter A (2000). Are microorganisms more effective than plants at competing for nitrogen?. Trends Plant Sci..

[48] Moreau D, Bardgett RD, Finlay RD, Jones DL, Philippot L (2019). A plant perspective on nitrogen cycling in the rhizosphere. Funct. Ecol..

[49] Kitzler B, Zechmeister-Boltenstern S, Holtermann C, Skiba U, Butterbach-Bahl K (2006). Nitrogen oxides emission from two beech forests subjected to different nitrogen loads. Biogeosciences.

[50] Guo X, Drury CF, Yang X, Daniel Reynolds W, Fan R (2014). The extent of soil drying and rewetting affects nitrous oxide emissions, denitrification, and nitrogen mineralization. Soil Sci. Soc. Am. J..

[51] Weier KL, Doran JW, Power JF, Walters DT (1993). Denitrification and the dinitrogen/nitrous oxide ratio as affected by soil water, available carbon, and nitrate. Soil Sci. Soc. Am. J..

[52] Liu T (2019). Earthworms coordinate soil biota to improve multiple ecosystem functions. Curr. Biol..

