# Supplementary material for: Earthworms do not increase greenhouse gas emissions (CO2 and N2O) in an ecotron experiment simulating a three-crop rotation system
Source: Sci Rep. 2023 Dec 8;13:21920. doi: 10.1038/s41598-023-48765-3 (PMC10713613; doi:10.1038/s41598-023-48765-3)
Supplement: Supplementary file 1 — Supplementary Information. [file 41598_2023_48765_MOESM1_ESM.docx]

Earthworms do not increase greenhouse gas emissions (CO_2_ and N_2_O) in an ecotron experiment simulating a three-crop rotation system

**Supplementary information file containing:**

1. **Supplementary methods**
2. **Supplementary results**
3. **Supplementary Tables S1-S5**
4. **Supplementary Figures S1-S7**
5. **Supplementary references**

**Supplementary methods**

*Simulated climatic conditions*

To account for the differences in solar radiation between Montpellier and the reference site, a shading black mesh was mounted on top of the transparent domes form October to March to bring the integrated solar radiation to similar levels; no difference was observed in summer when the 15% attenuation due to the Teflon-FEP film was sufficient. Global radiation (Rg) was measured every 20 s with a BF5 Sunshine Sensor (Delta-D device) located in a weather station close to the Macrocosms platform. Air temperature and relative humidity were measured every 20 s by a DT269 Duct Mount Digital Relative Humidity and Temperature Transmitter (MICHELL Instruments) located in the middle of each macrocosm in a solar shelter at 1.5 m aboveground. Volumetric soil moisture and soil temperature were measured every 5 min. with TDR sensors (Trime PICO 32, IMKO manufacturer) located at 0.13, 0.25 and 0.8 m depth and at three distances from the western edge of the lysimeter (0.65, 1.25 (lysimeter center) and 1.85m). Irrigation was performed at night and the amount of water applied with sprinkler systems was defined so as to mimic the amount of rainfall of the EFELE experimental site from which the soil was extracted. In addition, TDR soil moisture values at 0.13 and 0.25 m depth were used in comparison with counterpart measurements at the reference site to inform whether compensation irrigation would occasionally be needed in order to compensate for the increased evapotranspiration that can occur in confined conditions due to continuous air flow in the dome.

*Supplementary statistics*

For the weekly mean analysis, a mixed linear model of the form “response_variable~EW_BM+EW_T*Week” with the macrocosm ID as a random effect (random=~1|mac) was used for each of the six response variables (NEE, GPP, Reco, N_2_O, ET and WUE) using the lme() function from the nlme package (version 3.1-160)^1^. Following the guidelines suggested by^2^, in a first step we identified the most appropriate random structure using a restricted maximum likelihood approach by testing five different models for each response variable and then selecting the random structure with the lowest Akaike information criterion (AIC): Model 0 (reference), Model 1 (reference with autocorrelation structure “correlation = corAR1(form = ~1|mac)”), Model 2 (Model 1 with weighting structure for variances between weeks, “weights=varIdent(form=~1|Week)”), Model 3 (Model 1 with weighting structure for variances between EW_T levels, “weights=varIdent(form=~1|EW_T)”), and Model 4 (Model 1 with weighting structure for variances between the interaction of EW_T*Week, “weights=varIdent(form=~1|EW_T*Week)”). In a second step, model simplification to reach the minimal adequate model was performed on the model with the lowest AIC selected during the first step. This was done using the stepAIC() function from the MASS package (version 7.3-58.1)^3,4^. For the per crop cumulated analysis, the statistical procedure resembled the one followed for the weekly means, with the notable differences that the Week predictor is no-longer pertinent and was removed, all variables were log transformed to reduce the sensitivity of the models to data scarcity (less data points since values were cumulated), and that a generalized least squares model (gls) of the form gls(log(response_variable) ~ EW_T + EW_BM) was used for each of the six response variables. Only two models were compared using the same comparison procedure: (i) Model 1, gls(log(Response_variable)~EW_T+EW_BM) and (ii) model 2, model 1 with the addition of a weighting structure taking into account the possible differences in variances between EW_T levels (weights=varIdent(form=~1|EW_T)). For the whole crop sequence analysis, the same models and procedure as for the per crop cumulated analysis were used, this time on the whole crop sequence thus including the cumulated values from wheat, intercrop_1, mustard, intercrop_2 and maize crops. To denote differences between the means of cumulative values, Tukey’s post-hoc honest significant difference (HSD) test was used^5^. This test is commonly used in situations where multiple pairwise comparisons are made, and helps to control the overall false positive rate. However, as this test is not capturing all of the relevant sources of variation in the data, occasional discrepancies between the mixed-effects fitted coefficients and the Tukey test can occur. Given the inherent trade-offs between advanced measurement capabilities and the limited number of available experimental units in ecotrons^6^, we also address results that are marginally significant when the effect sizes are large ^7^.

**Supplementary results**

*Intercrop between the wheat and mustard crops (intercrop_1)*

NEE weekly fluxes values were negative during the whole intercrop, indicating a net CO_2_ release from the macrocosms to the atmosphere due to higher Reco than GPP during daytime in this period with very little vegetation (Fig. S1a). The NEE weekly fluxes were significantly affected by Week (P-value < 0.001; Table S4), and the EW_T and EW_B treatments, decreasing with EW_BM (P-value = 0.012; Table S4) and being increased by anecic earthworms (P-value = 0.031; Table S4). The cumulative NEE emissions reached -403.38 (±63.37), -366.90 (±33.85) and -343.66 (±65.95) g CO_2_ m^-2^ in the control, anecic and endogeic earthworm treatment levels, respectively, and were neither influenced by EW_T nor EW_BM. The GPP weekly fluxes (Fig. S1c) only varied with the experimental Week (P-value < 0.001; Table S4). The cumulative GPP fluxes reached 324.83 (±35.76), 375.67 (±37.66) and 428.96 (±50.43) g CO_2_ m^-2^ in the control, anecic and endogeic earthworm treatment levels, respectively, and were not influenced neither by the earthworm biomass nor by the earthworm treatment (Table S4; Fig. S1d).

Reco weekly emissions followed quite similar dynamics to GPP, showing a progressive decrease in absolute value with time (Fig. S1e), and were also influenced by Week (P-value < 0.001; Table S4). The earthworm treatment marginally significantly affected Reco (P-value = 0.062; Table S4), with a tendency of lower Reco values (i.e., indicating higher respiration) in both anecic and endogeic treatment levels relative to control. However, the cumulative Reco fluxes reached 753.30 (±29.89), 773.84 (±42.45) and 807.04 (±35.80) g CO_2_ m^-2^ in the control, anecic and endogeic earthworm treatment levels, respectively, and neither EW_T nor EW_BM showed any statistically significant effects (Table S4; Fig. S1f).

The weekly N_2_O emissions oscillated around 0.2 g m^-2^ day^-1^ in the control and anecic treatments and around 0.1 g m^-2^ day^-1^ in the endogeic earthworm treatment level from the beginning of the intercrop period until the beginning of October, where all levels converged around 0.2 g m^-2^ day^-1^ and progressively decreased henceforth (Fig. S1g). Weekly N_2_O emissions were significantly influenced by the EW_T×Week two-way interaction (P-value < 0.001; Table S4), the fitted interaction slopes indicating some weeks with decreased emissions relative to control at the beginning of the intercrop and increased emissions relative to control at the end of the intercrop only in the endogeic treatment level. The cumulative N_2_O fluxes reached 19.46 (±3.35), 19.96 (±6.01) and 10.12 (±1.83) g m^-2^ in the control, anecic and endogeic earthworm treatment levels, respectively, and showed a marginally statistically significant effect of EW_T (P-value = 0.07; Table S4), N_2_O emissions being significantly lower in the endogeic EW_T level, but the effect size is arguably large (-48%) relative to control (Fig. S1h).

Weekly ET fluxes followed the apparition and growth of weeds after the harvest of the previous culture, peaking twice at the beginning of August and the end of September, similar to the GPP fluxes (Fig. S1i). Weekly ET fluxes were significantly influenced by the EW_T×Week two-way interaction (P-value < 0.001; Table S4), the fitted interaction slopes indicating an increase in ET fluxes relative to control in both anecic and endogeic treatment levels mainly at the beginning and the end of the intercrop. Cumulative ET fluxes reached 208.89 (±4.24), 210.05 (±1.72) and 206.68 (±5.70) kg H_2_O m^-2^ in the control, anecic and endogeic earthworm treatment levels, respectively, and were found to decrease with earthworm biomass (P-value < 0.001; Table S4).

WUE rate varied throughout the intercrop period depending on the environmental conditions and the development of the weed with values between 0.2 and 7 g CO_2_ kg H_2_O^-1^ m^-2^ day^-1^ (Fig. S1k). WUE fluxes only significantly varied with Week (P-value < 0.001; Table S4). The cumulative WUE reached 265.61 (±35.07), 300.71 (±35.20) and 341.47 (±19.65) g CO_2_ kg H_2_O^-1^ m^-2^ in the control, anecic and endogeic earthworm treatment levels, respectively, and showed no statistically significant effects neither of EW_T nor EW_BM (Table S4; Fig. S1l).

*Intercrop between the mustard and maize crops (intercrop_2)*

The NEE weekly fluxes during this period were both negative and positive, depending on the evolution of the leftover crop biomass and weed development after the harvest (Fig. S2a). The NEE weekly fluxes were marginally influenced by EW_T (P-value = 0.09), with the NEE values being statistically marginally higher in the endogeic EW_T level relative to control, in addition to a Week effect (P-value < 0.001; Table S5). The cumulative NEE emissions reached -0.05 (±12.52), 13.12 (±24.92) and 35.53 (±14.60) g CO_2_ m^-2^ in the control, anecic and endogeic earthworm treatment levels, respectively, but the high variability observed within the EW_T levels led to no significant effects of EW_T nor EW_BM (Table S5; Fig. S2b). The GPP weekly fluxes (Fig. S2c) were only influenced by Week (P-value < 0.001; Table S5). The cumulative GPP fluxes reached 197.66 (±4.24), 237.13 (±42.31) and 299.89 (±42.56) g CO_2_ m^-2^ in the control, anecic and endogeic earthworm treatment levels, respectively, and showed a marginally statistically significant effect of EW_T (P-value = 0.0749; Table S5), the GPP emissions being significantly higher in the endogeic earthworm treatment level (+51.7%; Fig. S2d) relative to control.

Reco weekly emissions started to increase in absolute value for all EW_T levels at the beginning of April and reached in the end of April values four times as high as in late March, to slowly decrease henceforth (Fig. S2e). Reco weekly emissions were significantly affected by the EW_T×Week interaction (P-value = 0.001; Table S5) with a couple of weeks where both anecic and endogeic earthworms marginally increased Reco emissions. The cumulative Reco fluxes reached 215.86 (±15.46), 243.65 (±36.36) and 288.09 (±34.36) g CO_2_.m^-2^ in the control, anecic and endogeic earthworm treatment levels, respectively, and showed no statistically significant effects neither of EW_T nor EW_BM (Table S5; Fig. S2f).

The weekly N_2_O emissions oscillated around 0.4 g m^-2^ day^-1^ for all treatments (Fig. S2g) and were significantly influenced by the Week×EW_T interaction (P-value = 0.004; Table S5), the fitted interaction slopes indicating several weeks where the emissions were higher in the anecic treatment relative to control. The cumulative N_2_O fluxes reached 24.15 (±3.84), 19.11 (±0.46) and 21.76 (±0.35) g m^-2^ in the control, anecic and endogeic earthworm treatment levels, respectively and were found to decrease with earthworm biomass (P-value < 0.001; Table S5).

Weekly ET fluxes increased from 1 kg H_2_O m^-2^ day^-1^ to 2.5 the first month of the intercrop period while GPP emissions were decreasing during this time, indicating that soil evaporation was the main contributor to ET during this period, then plateaued henceforth (Fig. S2i). In addition to statistically significant weekly fluctuations (P-value < 0.001; Table S5), ET slightly decreased with earthworm biomass (P-value = 0.002; Table S5) but increased in the anecic treatment relative to control (P-value = 0.015; Table S5). Cumulative ET fluxes reached 114.23 (±1.88), 118.06 (±1.55) and 111.40 (±2.95) kg H_2_O m^-2^ in the control, anecic and endogeic earthworm treatment levels, respectively, and were not influenced by earthworm treatment or earthworm biomass (Table S5; Fig. S2j).

Weekly WUE rates showed a 50% decrease in all EW_T levels as the spontaneous vegetation started growing the first week of April and slightly increased until the end of April to decrease henceforth (Fig. S2k). Weekly WUE rates only varied with the experimental Week (P-value < 0.001; Table S5). The cumulative WUE reached 132.97 (±9.79), 125.73 (±14.56) and 170.72 (±9.57) g CO_2_ kg H_2_O^-1^ m^-2^ in the control, anecic and endogeic earthworm treatment levels, respectively, and showed a statistically significant EW_T effect (P-value = 0.031; Table S5) with cumulated WUE being significantly higher in the endogeic earthworm treatment level relative to control (+28%; Fig. S2l).

**References**

1. Pinheiro, J. C., Bates, D. J., DebRoy, S. & Sakar, D. *The Nlme Package: Linear and Nonlinear Mixed Effects Models, R Version 3*. *R package version* vol. 6 (2012).

2. Zuur, A. F., Ieno, E. N., Walker, N. J., Saveliev, A. A. & Smith, G. M. *Mixed Effects Models and Extensions in Ecology with R*. (Springer-Verlag, 2009).

3. Ripley, B. *et al.* MASS: Support Functions and Datasets for Venables and Ripley’s MASS. (2022).

4. Venables, W. N. & Ripley, B. D. *Modern Applied Statistics with S*. (Springer, 2002). doi:10.1007/978-0-387-21706-2.

5. Tukey, J. W. Comparing Individual Means in the Analysis of Variance. *Biometrics* **5**, 99 (1949).

6. Roy, J. *et al.* Ecotrons: Powerful and versatile ecosystem analysers for ecology, agronomy and environmental science. *Glob. Change Biol.* **27**, 1387–1407 (2021).

7. Wasserstein, R. L. & Lazar, N. A. The ASA Statement on p-Values: Context, Process, and Purpose. *Am. Stat.* **70**, 129–133 (2016).

**Supplementary tables**

**Table S1:** Experimental details of the crop sequence: sowing and harvest dates, as well as sowing density, for each crop.

| **Crop** | **Sowing date** | **Harvest/destruction date** | **Sowing density** |
| --- | --- | --- | --- |
| Winter wheat | 2017-11-29 | 2018-07-09 | 184 ind. m^-2^ |
| Intercrop 1 |  | 2018-11-21 | Bare ground with spontaneous weeds |
| Mustard | 2018-11-21 | 2019-03-29 | 1g m^-2^ (10 kg ha^-1^) |
| Intercrop 2 |  | 2019-05-16 | Bare ground with spontaneous weeds |
| Maize | 2019-05-16 | 2019-09-21 | 11 ind. m^-2^ |

**Table S2:** Spontaneous vegetation biomass (DW g m-2) in intercop_1 (between wheat and mustard) and intercrop_2 (between mustard and maize) weighted after manual weeding in each macrocosm. The manual weeding took place at the beginning of each intercrop.

| Treatment | Macrocosm | Biomass (g DW m-2) |
| --- | --- | --- |
| Intercrop_1 | | |
| ANECIC | 1 | 0 |
| ANECIC | 4 | 6.1775 |
| ANECIC | 5 | 6.16 |
| ANECIC | 7 | 7.835 |
| ENDOGEIC | 2 | 8.465 |
| ENDOGEIC | 6 | 9.8125 |
| ENDOGEIC | 10 | 4.1 |
| ENDOGEIC | 12 | 6.145 |
| CONTROL | 3 | 14.47 |
| CONTROL | 8 | 7.1325 |
| CONTROL | 9 | 1.1825 |
| CONTROL | 11 | 4.96 |
| Intercrop_2 | | |
| ANECIC | 1 | 6.94 |
| ANECIC | 4 | 4.704 |
| ANECIC | 5 | 34.67 |
| ANECIC | 7 | 36.186 |
| ENDOGEIC | 2 | 30.73 |
| ENDOGEIC | 6 | 73.094 |
| ENDOGEIC | 10 | 5.414 |
| ENDOGEIC | 12 | 22.782 |
| CONTROL | 3 | 31.13 |
| CONTROL | 8 | 32.57 |
| CONTROL | 9 | 4.592 |
| CONTROL | 11 | 4.086 |

**Table S3:** Physico-chemical properties of the anaerobic digestate used as fertilizer for the wheat crop.

| Physico-chemical parameters | | | |
| --- | --- | --- | --- |
| Parameter | Unit | on dry basis | on raw basis |
| Humidity | % |  | 92.3 |
| Dry matter | % |  | 7,7 |
| Volatil Matter | % | 62.5 | 4.8 |
| Mineral Matter | % | 37.5 | 2.9 |
| Organic carbon | % | 31.2 | 2.4 |
| Conductivity | µS cm |  | 5.28 |
| pH at 25°C | pH Unit |  | 8.5 |
| Nitrogen | | | |
| Kjeldahl Nitrogen | % N | 6.16 | 0.474 |
| Ammoniacal Nitrogen | % N | 1.82 | 0.14 |
| Organic Nitrogen | % N | 4.34 | 0.334 |
| C/N |  |  | 5.1 |
| Other elements | | | |
| Phosphorous | % P_2_O_5_ | 3.02 | 0.23 |
| Potassium | % K_2_O | 9.07 | 0.7 |
| Calcium | % CaO | 3.5 | 0.27 |
| Magnesium | % MgO | 1.58 | 0.12 |
| Sulfur | % SO_3_ | 1.97 | 0.15 |
| Sodium | % Na_2_O | 0.43 | 0.033 |
| Equivalent g kg^-1^ or kg ton^-1^ | | | |
| Dry matter | g kg^-1^ |  | 76.6 |
| Volatil Matter | g kg^-1^ | 624.6 | 48.1 |
| Kjeldahl Nitrogen | g kg^-1^ | 61.6 | 4.74 |
| Ammoniacal Nitrogen | g kg^-1^ | 18.2 | 1.4 |
| Organic Nitrogen | g kg^-1^ | 43.4 | 3.34 |
| Phosphorous | g kg^-1^ | 30.2 | 2.3 |
| Potassium | g kg^-1^ | 90.7 | 7 |
| Calcium | g kg^-1^ | 35 | 2.7 |
| Magnesium | g kg^-1^ | 15.8 | 1.2 |
| Sulfur | g kg^-1^ | 19.7 | 1.5 |
| Sodium | g kg^-1^ | 4.3 | 0.33 |

**Table S4**: Minimal adequate models (F-values) for (i) weekly time series as affected by the sampling week (Week), earthworm biomass (EW_B), treatment (EW_T) and their interactions, and (ii) cumulative emissions as affected by the earthworm biomass (EW_B) and treatment (EW_T) **in the intercrop between wheat and mustard**. “NA” stands for non-applicable. ***P < 0.001; **P < 0.01; *P< 0.05; +P < 0.1.

| Weekly time series | | | | | | |
| --- | --- | --- | --- | --- | --- | --- |
| Source | NEE | GPP | Reco | N_2_O | ET | WUE |
| Week | **F_19/209_ = 274.03***** | **F_19/209_ = 26.86***** | **F_19/209_ = 110.21***** | **F_18/162_ = 71.45***** | **F_19/171_ = 440.21***** | **F_19/209_ = 115.75***** |
| EW_BM | **F_1/8_ = 10.34***** | NA | F_1/8_ = 0.55 | NA | F_1/8_ = 1.29 | NA |
| EW_T | **F_2/8_ = 31.19***** | NA | F_2/8_ = 4 | F_2/9_ = 2.61 | **F_2/8_ = 6.59***** | NA |
| EW_T×Week | NA | NA | NA | **F_36/162_ = 3.03***** | **F_38/171_ = 2.26***** | NA |
| Cumulative | | | | | | |
| EW_BM | NA | NA | NA | F_1/8_ = 2.62 | **F_1/8_ = 28.68***** | NA |
| EW_T | NA | NA | NA | F_2/8_ = 3.78+ | F_2/8_ = 0.59 | NA |

**Table S5**: Minimal adequate models (F-values) for (i) weekly time series as affected by the sampling week (Week), earthworm biomass (EW_B), treatment (EW_T) and their interactions, and (ii) cumulative emissions as affected by the earthworm biomass (EW_B) and treatment (EW_T) **in the intercrop between mustard and maize**. “NA” stands for non-applicable. ***P < 0.001; **P < 0.01; *P< 0.05; +P < 0.1.

| Weekly time series | | | | | | |
| --- | --- | --- | --- | --- | --- | --- |
| Source | NEE | GPP | Reco | N_2_O | ET | WUE |
| Week | **F_7/77_ = 50.32***** | **F_7/77_ = 8.07***** | **F_7/63_ = 311.67***** | **F_4/36_ = 177.78***** | **F_7/77_ = 144.11***** | **F_7/77_ = 15.78***** |
| EW_BM | NA | NA | NA | **F_1/8_ = 66.83***** | **F_1/8_ = 20.07***** | F_1/8_ = 0.12 |
| EW_T | F_2/9_ = 2.44 | NA | F_2/9_ = 1.13 | **F_2/8_ = 20.23***** | **F_2/8_ = 7.46***** | F_2/8_ = 2.04 |
| EW_T$\times$Week | NA | NA | **F_14/63_ = 3.03***** | **F_8/36_ = 3.57***** | NA | NA |
| Cumulative | | | | | | |
| EW_BM | NA | NA | NA | **F_1/10_ = 92.61***** | NA | NA |
| EW_T | NA | F_2/9_ = 3.5+ | NA | NA | F_2/9_ = 2.21 | **F_2/9_ = 5.19***** |

**Supplementary figures**


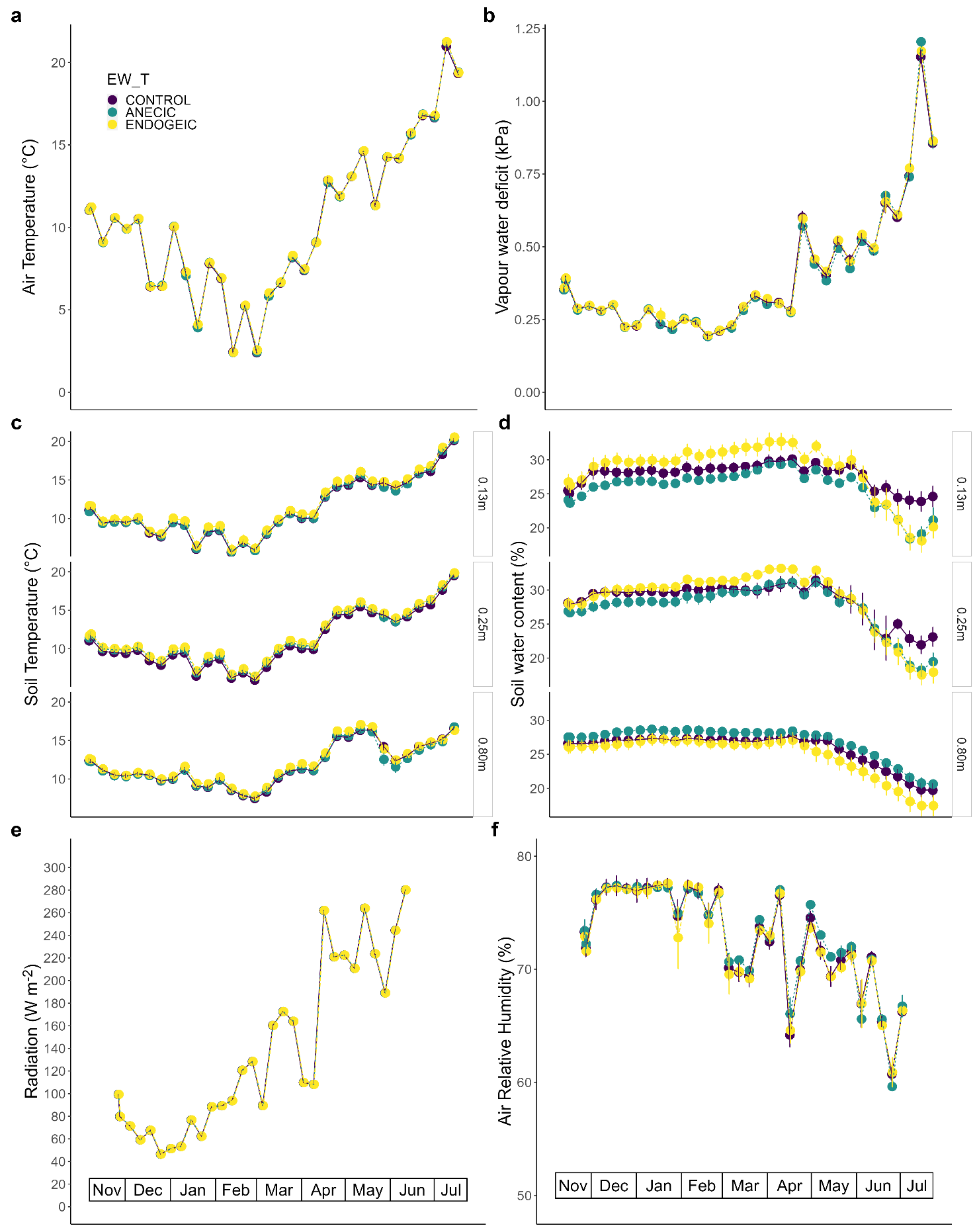


**Figure S1:** Weekly dynamics of environmental variables as affected by the earthworm treatment (EW_T) **in the wheat crop**. (**a**) Radiation (averaged over 24h). (**b**) Air humidity. (**c**) Air temperature. (**d**) VPD. (**e**) Soil temperature. (**f**) Soil water content. Data represent means ± SEM of four replicates. Horizontal bottom bars represent the experimental periods (crop growing season).


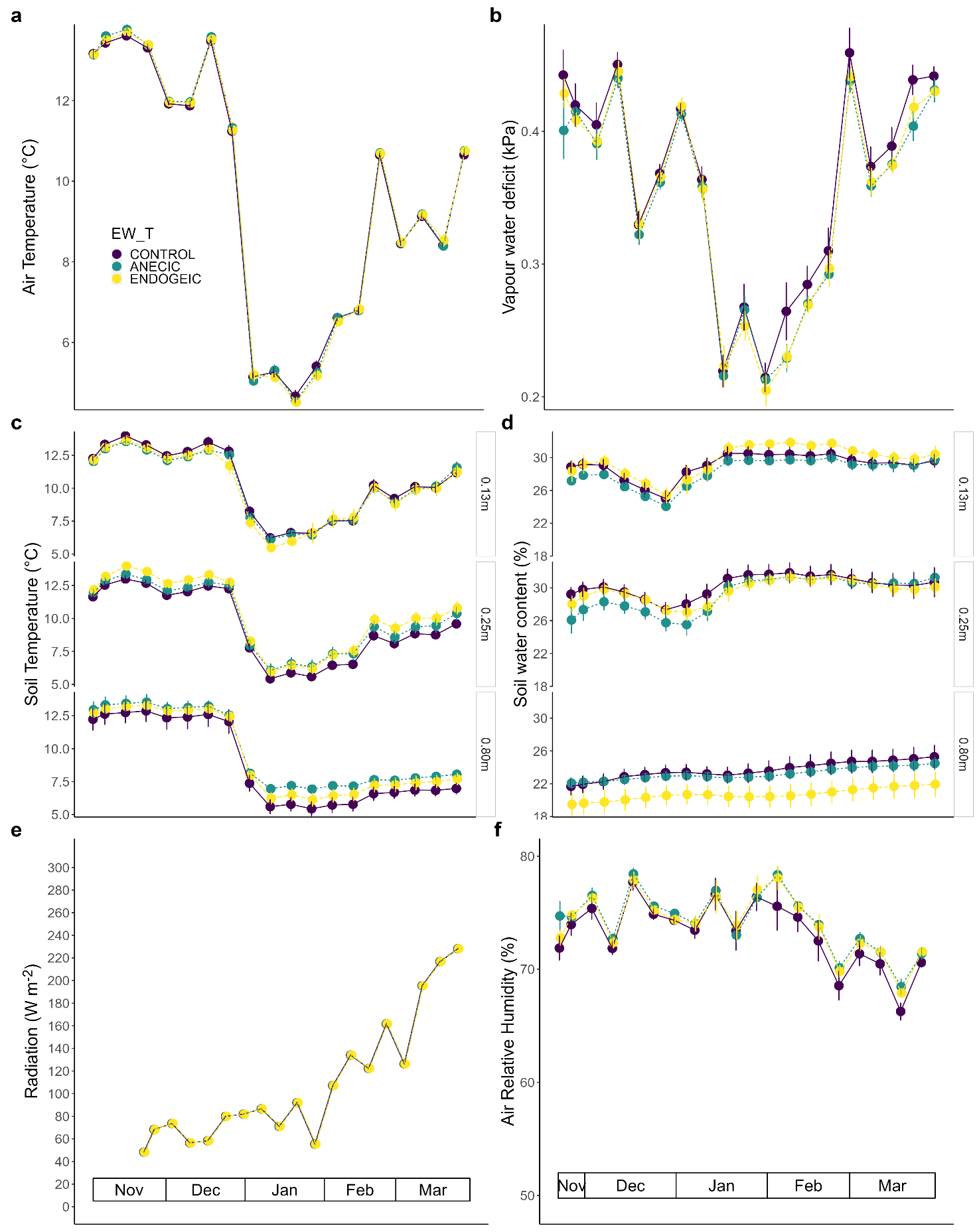


**Figure S2:** Weekly dynamics of environmental variables as affected by the earthworm treatment (EW_T) **in the mustard crop**. (**a**) Radiation (averaged over 24h). (**b**) Air humidity. (**c**) Air temperature. (**d**) VPD. (**e**) Soil temperature. (**f**) Soil water content. Data represent means ± SEM of four replicates. Horizontal bottom bars represent the experimental periods (crop growing season).


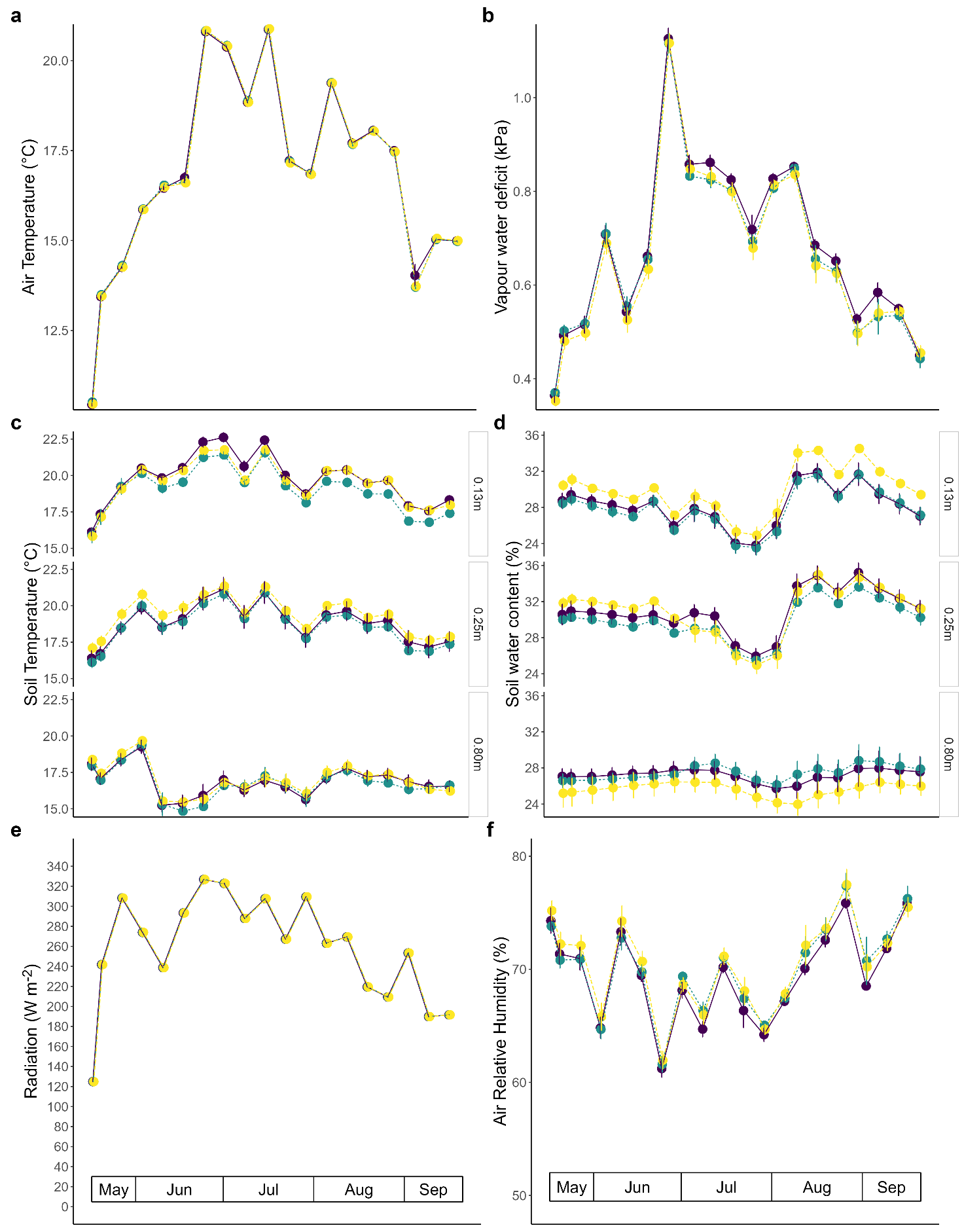


**Figure S3**: Weekly dynamics of environmental variables as affected by the earthworm treatment (EW_T) **in the maize crop**(**a**) Radiation (averaged over 24h). (**b**) Air humidity. (**c**) Air temperature. (**d**) VPD. (**e**) Soil temperature. (**f**) Soil water content. Data represent means ± SEM of four replicates. Horizontal bottom bars represent the experimental periods (crop growing season).


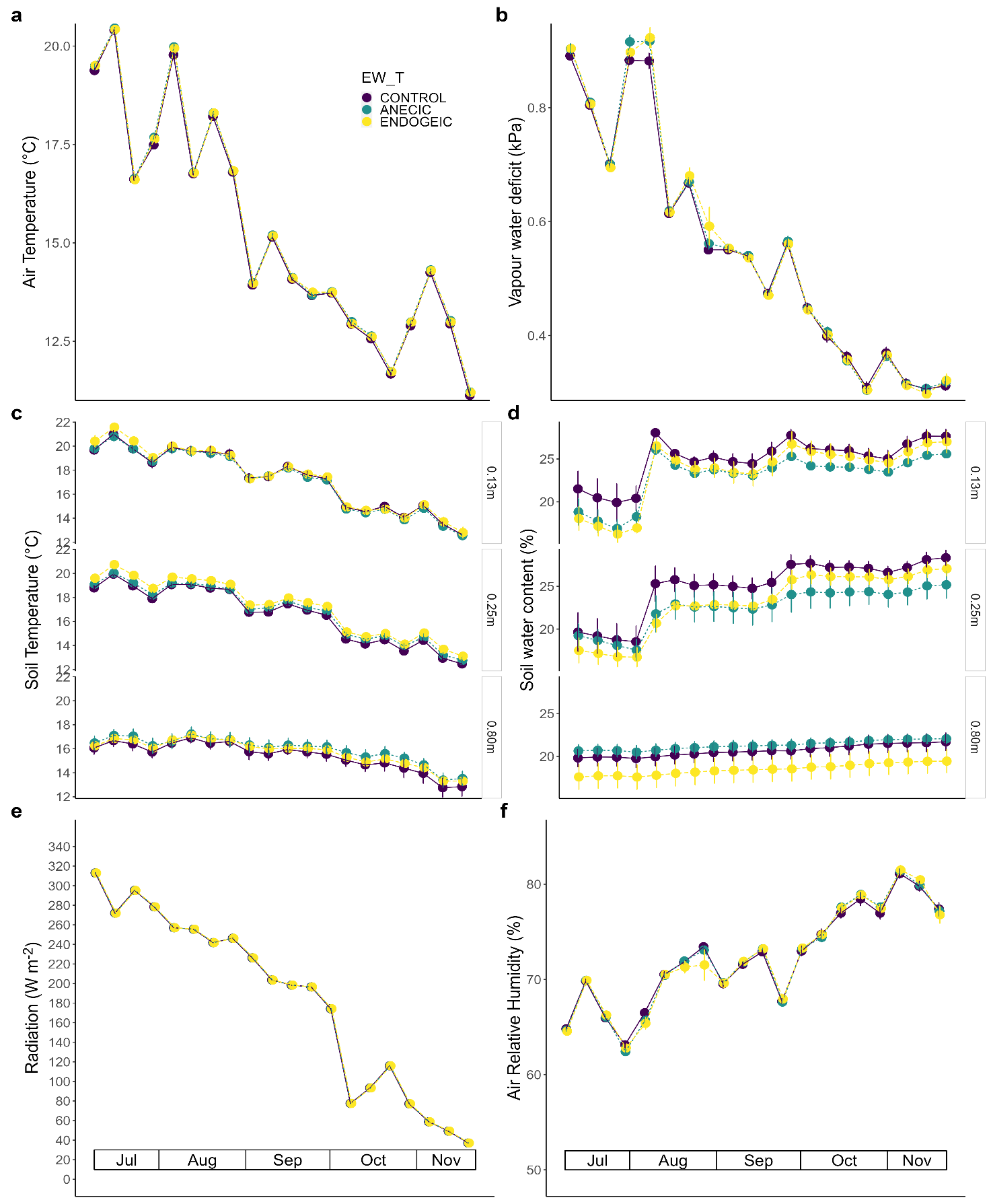


**Figure S4:** Weekly dynamics of environmental variables as affected by the earthworm treatment (EW_T) in the **in the intercrop between wheat and mustard.** (**a**) Radiation (averaged over 24h). (**b**) Air humidity. (**c**) Air temperature. (**d**) VPD. (**e**) Soil temperature. (**f**) Soil water content. Data represent means ± SEM of four replicates. Horizontal bottom bars represent the experimental periods (crop growing season).


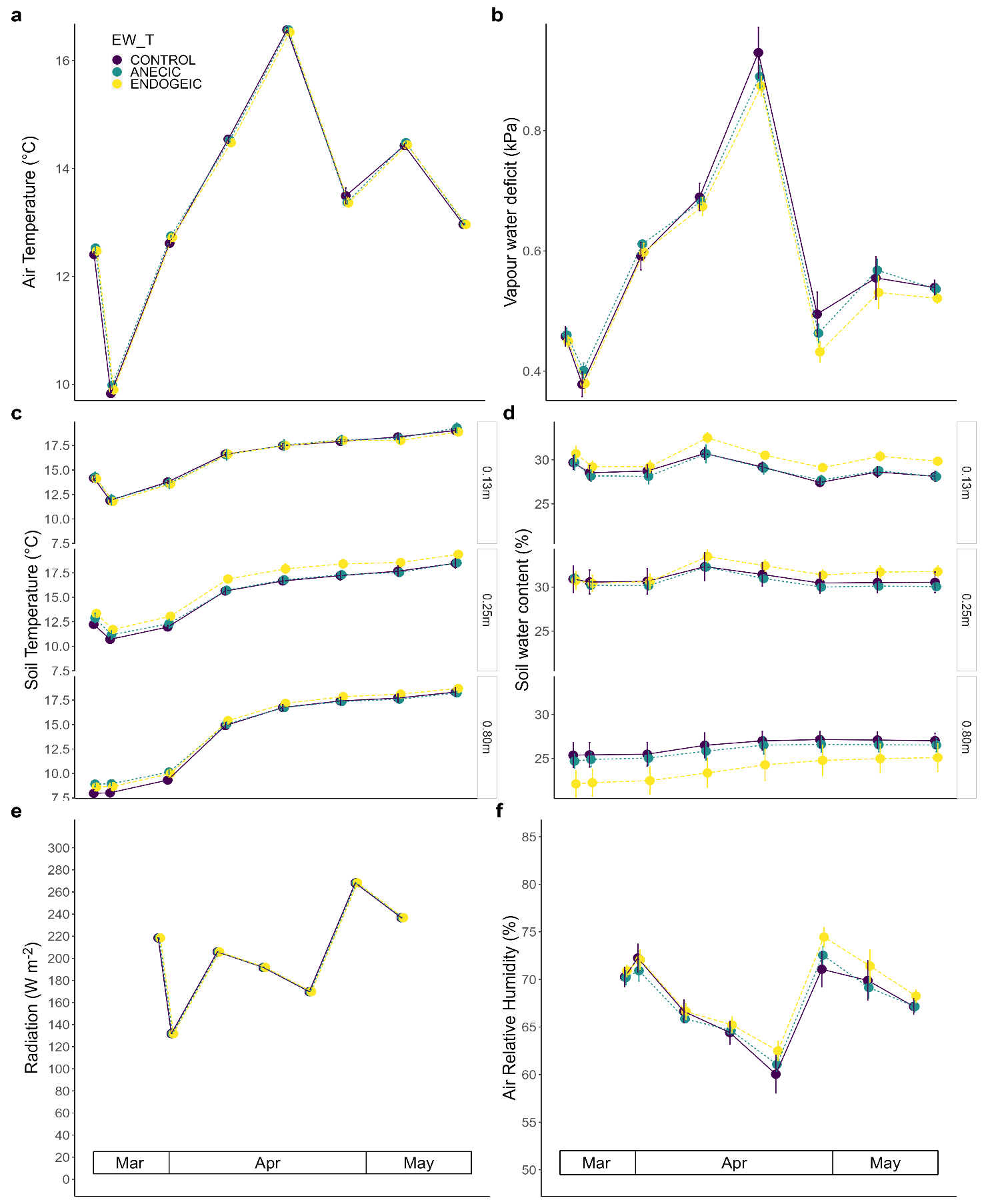


**Figure S5**: Weekly dynamics of environmental variables as affected by the earthworm treatment (EW_T) in the **in the intercrop between mustard and maize**. (**a**) Radiation (averaged over 24h). (**b**) Air humidity. (**c**) Air temperature. (**d**) VPD. (**e**) Soil temperature. (**f**) Soil water content. Data represent means ± SEM of four replicates. Horizontal bottom bars represent the experimental periods (crop growing season).


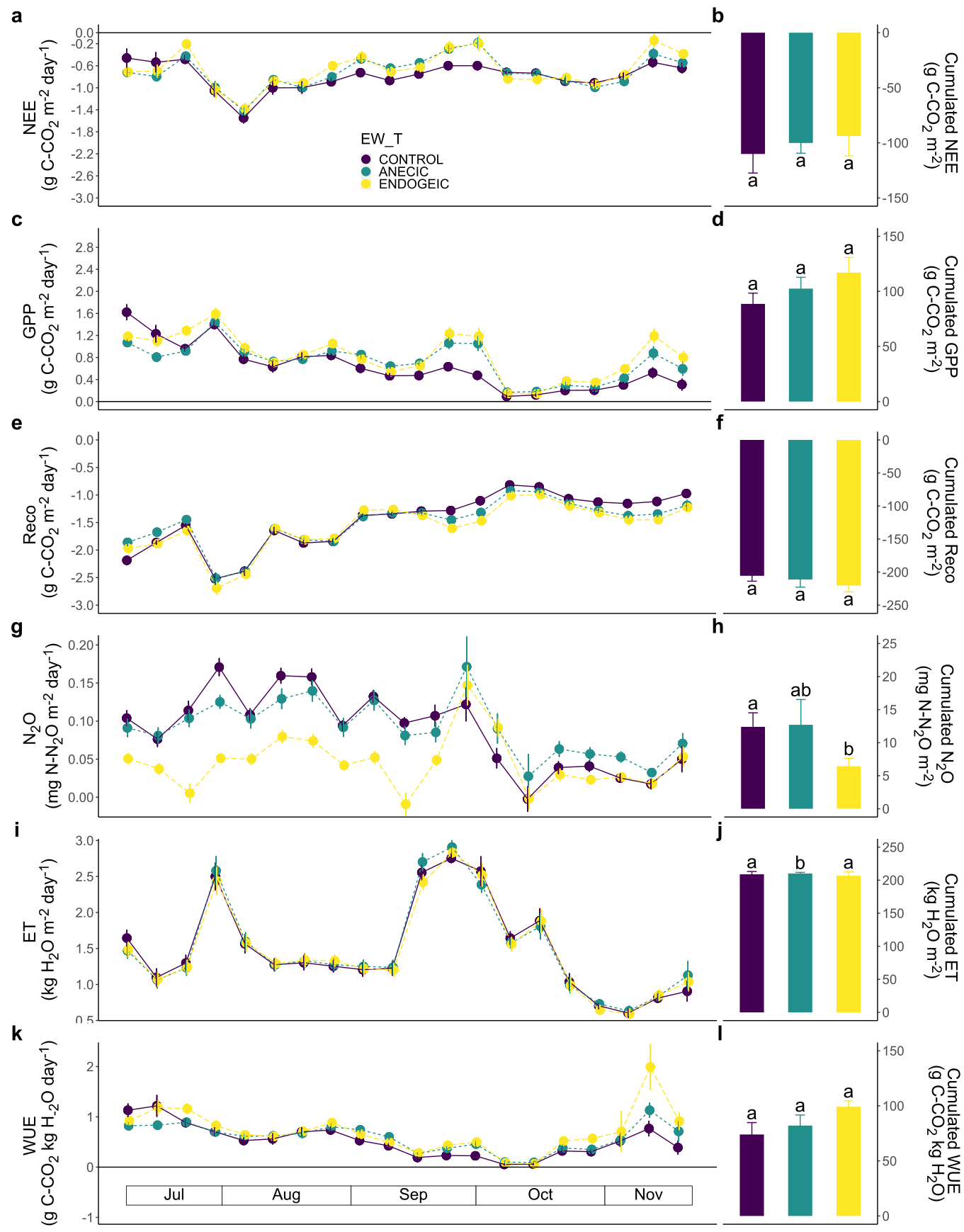


**Figure S6:** Weekly dynamics (Left, lines) and cumulative fluxes (Right, bars) of carbon, N_2_O and water as affected by the earthworm treatment (EW_T) **in the intercrop between wheat and mustard**. (**a, b**) NEE. (**c, d**) GPP. (**e, f**) Reco. (**g, h**) N_2_O. (**i, j**) ET. (**k, l**) WUE. Data represent means ± SEM of four replicates. Different letters above bars denote significant differences between individual means. Horizontal bottom bars represent the experimental periods (crop growing season).


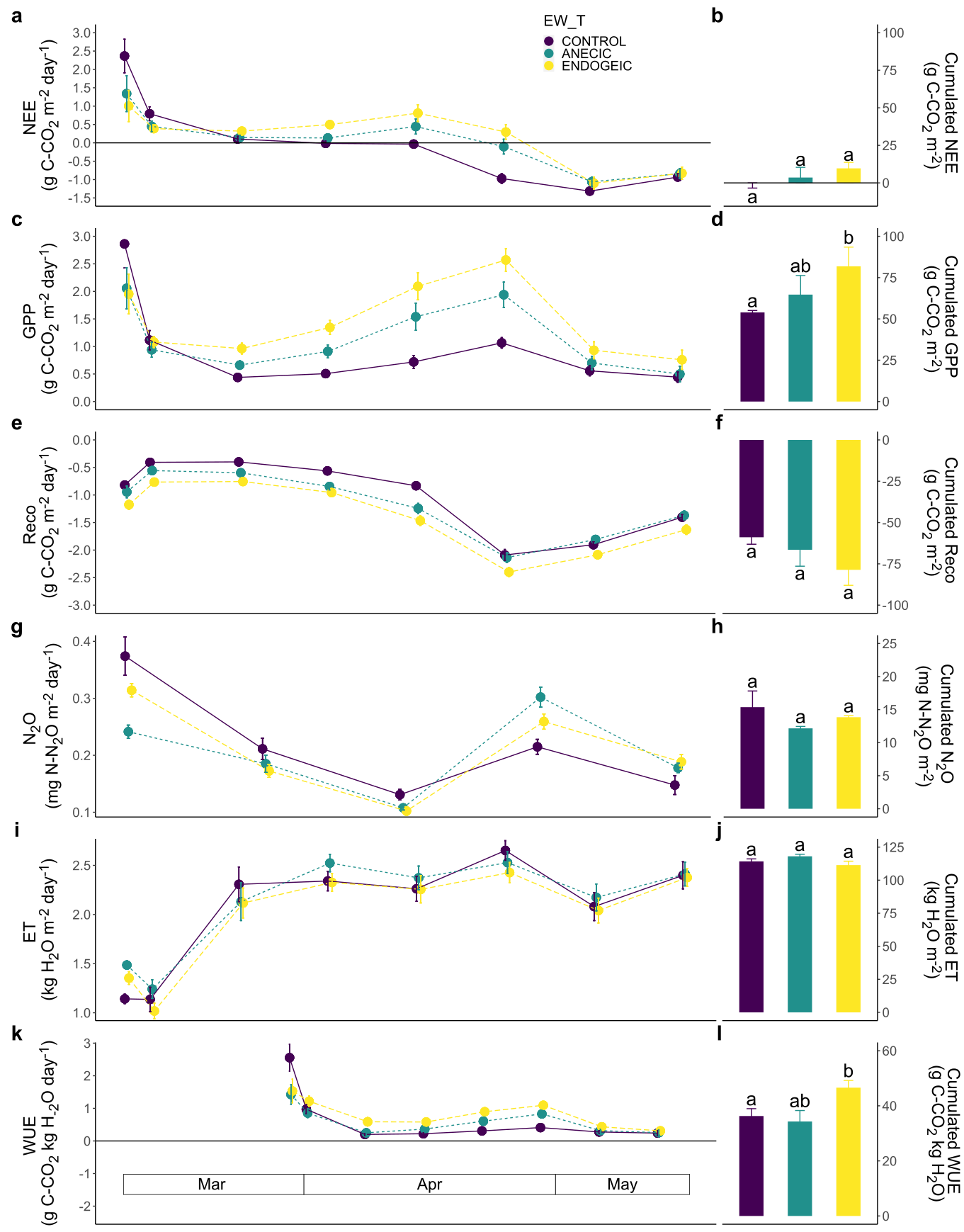


**Figure S7:** Weekly dynamics (Left, lines) and cumulative fluxes (Right, bars) of carbon, N_2_O and water as affected by the earthworm treatment (EW_T) **in the intercrop between mustard and maize**. (**a, b**) NEE. (**c, d**) GPP. (**e, f**) Reco. (**g, h**) N_2_O. (**i, j**) ET. (**k, l**) WUE. Data represent means ± SEM of four replicates. Different letters above bars denote significant differences between individual means. Horizontal bottom bars represent the experimental periods (crop growing season).
